# METTL5 Enables Immune Evasion of Liver Cancer via Chemokine mRNA Translation Regulation

**DOI:** 10.1002/advs.202512528

**Published:** 2025-12-23

**Authors:** Shuang Li, Xiao Zhao, Tongtong Song, Qiaoyi Chen, Yanqing Wu, Yuting Zhang, Jingying Chen, Yifan Wu, Bin Li, Xinyue Zhang, Zihao Dai, Lixia Xu, Yubin Xie, Alfred Sze‐Lok Cheng, Jianping Guo, Ming Kuang, Shuibin Lin, Zhenwei Peng, Sui Peng, Xuezhen Zeng

**Affiliations:** ^1^ Department of Liver Surgery Center of Hepato‐Pancreato‐Biliary Surgery The First Affiliated Hospital Sun Yat‐sen University Guangzhou Guangdong 510080 China; ^2^ Department of Radiation Oncology Cancer Center The First Affiliated Hospital of Sun Yat‐sen University Guangzhou Guangdong 510080 China; ^3^ Department of Oncology The First Affiliated Hospital of Sun Yat‐sen University Guangzhou Guangdong 510080 China; ^4^ Department of Gastroenterology and Hepatology The First Affiliated Hospital Sun Yat‐sen University Guangzhou Guangdong 510080 China; ^5^ Department of Pathology The First Affiliated Hospital Sun Yat‐sen University Guangzhou Guangdong 510080 China; ^6^ Clinical Trials Unit The First Affiliated Hospital Sun Yat‐sen University Guangzhou Guangdong 510080 China; ^7^ Institute of Precision Medicine the First Affiliated Hospital Sun Yat‐sen University Guangzhou Guangdong 510080 China; ^8^ School of Biomedical Sciences The Chinese University of Hong Kong Hong Kong 999077 China; ^9^ Center for Translational Medicine The First Affiliated Hospital Sun Yat‐sen University Guangzhou Guangdong 510080 China

**Keywords:** anti‐tumor immunity, METTL5, mRNA translation, rRNA m^6^A modification

## Abstract

The liver microenvironment is essential to immune surveillance and liver cancer progression. Here, the aim is to identify the role of METTL5, the 18S rRNA m^6^A methyltransferase, in regulating the liver immune microenvironment to promote cancer progression. Liver‐specific *Mettl5* knockout (cKO) in mice exhibits increased immune cell infiltration, especially CD3^+^ and CD4^+^ T cells. Loss of *Mettl5* inhibits intrahepatic cholangiocarcinoma (ICC) progression. By scRNA‐seq analysis, it is found that ICC from both cKO mice and human METTL5 low expression group correlates with increased CD8^+^ T cells but decreased macrophages, which is associated with better survival. Adoptive transfer of macrophages significantly promotes ICC progression. scRNA‐seq and scTCR‐seq analysis show that cKO mice exhibit reduced immunosuppressive Ms4a7^+^C1qa^+^ tumor‐associated macrophages (TAMs) but increased intratumoral IFN‐γ^+^CD8^+^ T cell infiltration and expansion. Mechanistically, METTL5‐mediated 18S rRNA m^6^A modification downregulates the mRNA translation of *CXCL16* to exclude CD8^+^ T cells. Knockout of *Mettl5* significantly increases CD8^+^ T cell infiltration in vivo. Combined METTL5 targeting using lipid nanoparticle‐encapsulated siRNA and PD‐1 blockade provokes anti‐tumor immunity to eradicate ICC tumors. Additionally, METTL5^Low^ human ICC correlates with responsiveness to immunotherapy. The study highlights the strong immuno‐evasive ability of METTL5 as a promising therapeutic target in ICC.

## Graphical abstract

METTL5 reshapes the tumor immune microenvironment through ribosome 18S rRNA m6A modification to regulate the translation of chemokine mRNA. Targeting METTL5‐mediated immunosuppression unleashes anti‐tumor immunity and improves the efficacy of anti‐PD‐1 therapy.

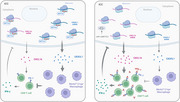

## 1 Introduction

Primary liver cancer (e.g., hepatocellular carcinoma, HCC; intrahepatic cholangiocarcinoma, ICC) remains a global health challenge.^[^ ^1^ ^]^ Liver cancer is the fifth leading cause of cancer‐related mortality and the incidence is still rising.^[^ ^2^, ^3^ ^]^ Although screening has improved survival through earlier diagnosis, the survival rate of liver cancer is still dismal.^[^ ^2^, ^3^ ^]^ Surgical resection is the first‐line treatment for early liver cancer, but only a small proportion of patients are eligible for curative resection,^[^ ^4^ ^]^ and the 5‐year recurrence rate is as high as 50–70%.^[^ ^5^, ^6^ ^]^ In the past decade, immune‐checkpoint inhibitors (ICIs) have revolutionized the management of cancers.^[^ ^7^ ^]^ However, the response rate is less than 20% in liver cancer,^[^ ^2^ ^]^ and the strong immunosuppressive microenvironment inhibits cytotoxic T cell infiltration and function, leading to low responsiveness to ICIs.^[^ ^8^, ^9^, ^10^ ^]^

The development, progression, and metastasis of tumor depend on the liver microenvironment.^[^ ^11^ ^]^ The liver is an essential organ of the body that functions in detoxifying various substances in the blood. The long‐termed exposure to toxins, viruses, or other substances that could lead to damage contributes to the immunosuppressive orientation of the liver.^[^ ^12^, ^13^, ^14^ ^]^ A large number of immune cells including Kupffer cells, macrophages, natural killer cells, T cells and B cells, accumulate in the liver and maintain homeostasis. The immune cells are the pivotal components that can both elicit anti‐tumor immune response and mediate immune tolerance.^[^ ^15^ ^]^ In conditions of liver damage or diseases, molecular danger patterns or inflammasome activation induce inflammatory responses leading to the chemokine‐mediated hepatic infiltration of circulating leukocytes.^[^ ^16^ ^]^ The mechanisms of how these changes alter immunosurveillance and thus influence the development of liver cancer are incompletely understood.

METTL5 has been identified as an enzyme responsible for catalyzing m^6^A modification at A‐1832 position on 18S rRNA that is proximate to the decoding center.^[^ ^17^ ^]^ The m^6^A_1832_ is involved in fine‐tuning the structural confirmation of the decoding center to promote translation.^[^ ^18^ ^]^ Multiple evidence showed that METTL5‐mediated dysregulation of mRNA translation is closely associated with cancer development. METTL5 promotes translation initiation by activating p70‐S6K, which supports breast cancer cell growth.^[^ ^18^ ^]^ In liver cancer, METTL5 establishes an oncogenic network to fuel tumor cell proliferation and metastasis.^[^ ^19^, ^20^, ^21^ ^]^ Mechanistically, METTL5 rewires glucose and fatty acid metabolism to promote tumor progression by translationally regulating USP5 that modulates the ubiquitination of c‐Myc and thus activated its downstream glycolytic genes,^[^ ^19^ ^]^ and by upregulating ACSL4 to enhance fatty acid metabolism.^[^ ^20^ ^]^ In addition, METTL5 also activates transforming growth factor (TGF)‐β pathway to enhance tumor metastasis.^[^ ^21^ ^]^ These studies highlight the pivotal role of METTL5 in cancer development and metastasis. However, whether and how METTL5‐mediated translation regulation in the liver immune microenvironment remains elusive.

In the present study, we unravel that METTL5 rewires the liver immune microenvironment to facilitate ICC progression. We further demonstrate that METTL5 modulates T cell infiltration through translational regulation of chemokine mRNA. The combination of METTL5 knockdown and anti‐PD‐1 therapy provoked anti‐tumor immunity to eradicate ICC tumors. Additionally, low METTL5 expression correlated with responsiveness to immunotherapy. This study uncovers that METTL5 shapes the immunosuppressive liver immune microenvironment for liver cancer progression and provides a novel target for ICC treatment and improving PD‐1 blockade efficacy.

## 2 Results

### 2.1 METTL5 is Associated with Immunotherapy Efficacy and Prognosis in Human ICC

**Figure 1:**
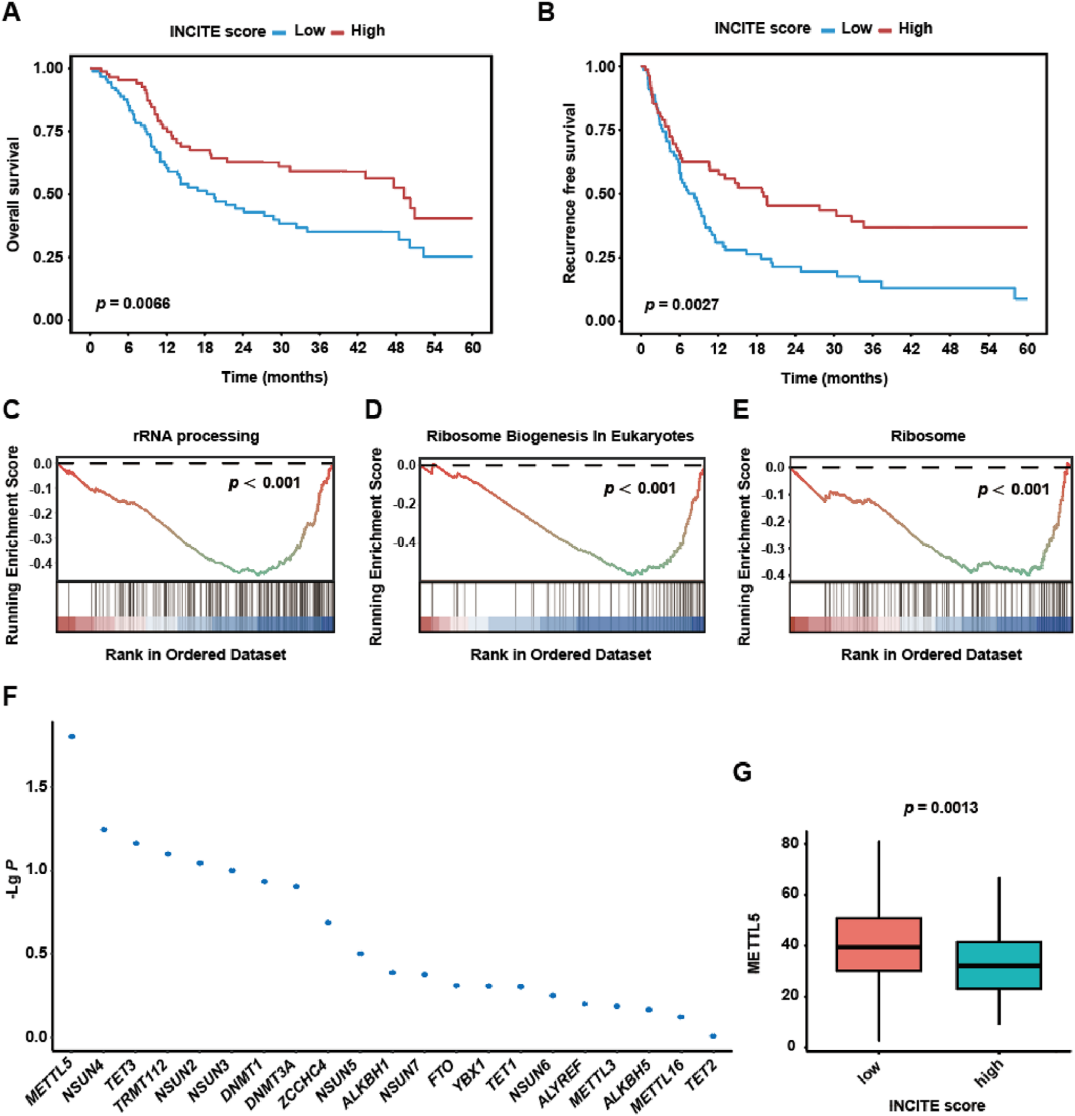
METTL5 is associated with immunotherapy efficacy and prognosis in human ICC. A total of 186 ICC bulk RNA‐seq data were separated into high and low INCITE score^[^ ^22^ ^]^ groups based on the median score. (n = 93 vs 93). A,B) The overall survival (A) and recurrence free survival (B) of 186 ICC patients. C–E) GSEA of rRNA processing (C), Ribosome Biogenesis In Eukaryotes (D), and Ribosome pathways (E). F) The differentially expressed genes that involved in ribosome and rRNA functions. G) The expression of *METTL5* in high and low INCITE score groups.

To explore the potential genes associated with immunotherapy efficacy, we analyzed a total of 186 ICC bulk RNA‐seq data from our cohort. The 186 ICC were separated into two groups based on the median of the INCITE score^[^ ^22^ ^]^ whose upregulation correlates with ICI‐induced tumor shrinkage. We found that a high INCITE score correlated with prolonged overall survival (OS) and recurrence free survival (RFS) (Figure **1**A,B). Gene set enrichment analysis (GSEA) showed that rRNA processing, Ribosome Biogenesis In Eukaryotes and Ribosome pathways were downregulated in high INCITE score group (Figure 1C–E). We further explored the expression of genes that are involved in ribosome and rRNA functions in two groups. It showed that METTL5, the 18S rRNA m^6^A methytransferase, was downregulated in high INCITE score group, indicating its negative correlation with ICB efficacy (Figure 1F,G).

### 2.2 METTL5 Shapes the Liver Immune Microenvironment to Promote Liver Cancer Progression

**Figure 2:**
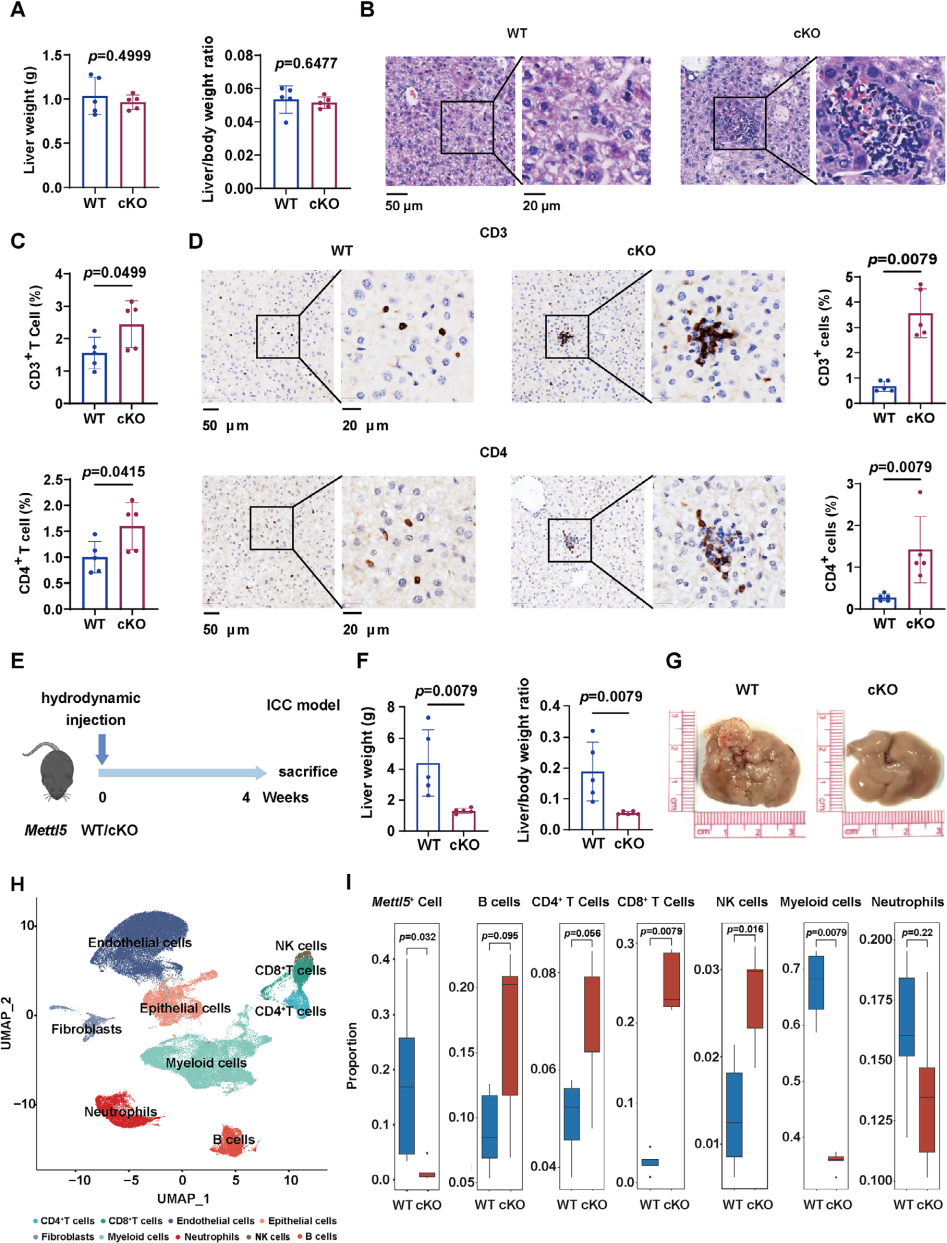
*Mettl5* shapes the liver immune microenvironment for tumor progression. A) Liver weight and Liver/body weight ratio of WT and cKO mice (n = 5). B) H&E staining of the mice livers. C) Flow cytometry analysis of CD3^+^, CD4^+^ T cells of the mice livers. D) IHC staining and statistic analysis of CD3 and CD4 in the livers of the mice. E) Schematic diagram of the experimental design. 3–4 weeks old *Mettl5* WT and cKO mice were hydrodynamically injected with YapS127A, AKT, and Sleeping beauty plasmids to induce ICC establishment. F) Liver weight and Liver/body weight ratio of WT and cKO mice (n=5). G) Representative gross images of the mice livers. H) scRNA‐seq was performed on livers from 5 WT and 5 cKO mice. Based on lineage marker expression, cells were grouped into 9 clusters and illustrated as a UMAP plot (n = 5). I) The proportion of *Mettl5^+^* cells in Epithelial cell cluster, B cells, CD4^+^ T cells, CD8^+^ T cells, NK cells, myeloid cells, and neutrophils in WT and cKO mice.

To investigate the potential role of METTL5 in the liver immune microenvironment, we employed a conditional hepatocyte‐specific *Mettl5* knockout (cKO) mouse model and found that knockout of *Mettl5* in the liver did not affect liver weight and liver weight to body weight ratio (Figure **2**A). Interestingly, we observed that the immune cell infiltration was increased in cKO mice livers compared to wild‐type (WT) mice livers (Figure 2B). We then performed flow cytometry analysis to detect liver infiltration of different immune cells, and illustrated that CD3^+^ T cells and CD4^+^ T cells were significantly increased in cKO mice livers, which was further validated by IHC staining (Figure S1 and S2A–H, Supporting Information; Figure 2C,D). In addition, no liver injury was observed upon *Mettl5* knockout (Figure S2I–K, Supporting Information).

Next, we explore whether METTL5‐mediated alteration of the liver immune microenvironment influenced tumor progression. Since there is no commercial mouse ICC cell line, we previously established a primary mouse ICC cell line (LTP‐C9)^[^ ^23^ ^]^ and enhanced the tumorigenicity of these cells by serial orthotopic implantation into C57BL/6 mice (Figure S2L, Supporting Information). Then, we constructed control and *Mettl5* knockout LTP‐C9 (G6) cells and orthotopically injected these cells to C57BL/6 mice. Furthermore, we also established ICC in WT and cKO mice by hydrodynamically injection of AKT, YAP, and Sleeping beauty plasmids. Intriguingly, we found that knockout of *Mettl5* significantly suppressed ICC development in both two models (Figure 2E–G; Figure S2M, Supporting Information). To elucidate the contribution of *Mettl5*‐mediated tumor and immune regulation in tumor progression, we performed CCK8 assay in LTP‐C9 (G6) cells and showed that knockout of *Mettl5* inhibited tumor cell growth (Figure S2N, Supporting Information). We also implanted sgNC and sg*Mettl5* LTP‐C9 (G6) cells into immune‐deficient NCG mice (Figure S2O, Supporting Information). Interestingly, we observed that *Mettl5* depletion reduced tumor burden by 71 and 74%, respectively, in immune‐competent mouse models (Figure 2F; Figure S2M, Supporting Information) while reduced tumor burden by 46% in immune‐deficient mice (Figure S2O, Supporting Information), indicating that the reshaping of liver immune microenvironment also plays a pivotal role in METTL5‐mediated ICC progression.

Then, we isolated and dissociated livers from WT and cKO mice of the hydrodynamic injection ICC model (n = 5 per group) for single cell RNA sequencing (scRNA‐seq). After initial quality control, a total of 25396 cells with a median of 924 genes detected per cell were used for Uniform Manifold Approximation and Projection (UMAP) dimensionality reduction and unsupervised graph‐based clustering. Nine populations were identified based on the expression of lineage marker genes, including CD4^+^ T cells, CD8^+^ T cells, endothelial cells, epithelial cells, fibroblasts, myeloid cells, neutrophils, NK cells and B cells (Figure 2H; Figure S2P, Supporting Information). Of them, *Mettl5* was successfully knockout in epithelial cells (Figure 2I). We next focused on the immune cell populations and uncovered that myeloid cells were dramatically decreased, while CD8^+^ T cells were increased in cKO mice (Figure 2I). The proportion of immune cells was further validated by flow cytometry analysis (Figure S2Q–V, Supporting Information). These data demonstrate that METTL5 shapes the liver immune microenvironment to facilitate ICC progression.

### 2.3 High METTL5 Expression in Human ICC Tumors is Associated with Immunosuppressive Microenvironment and Poor Prognosis

**Figure 3:**
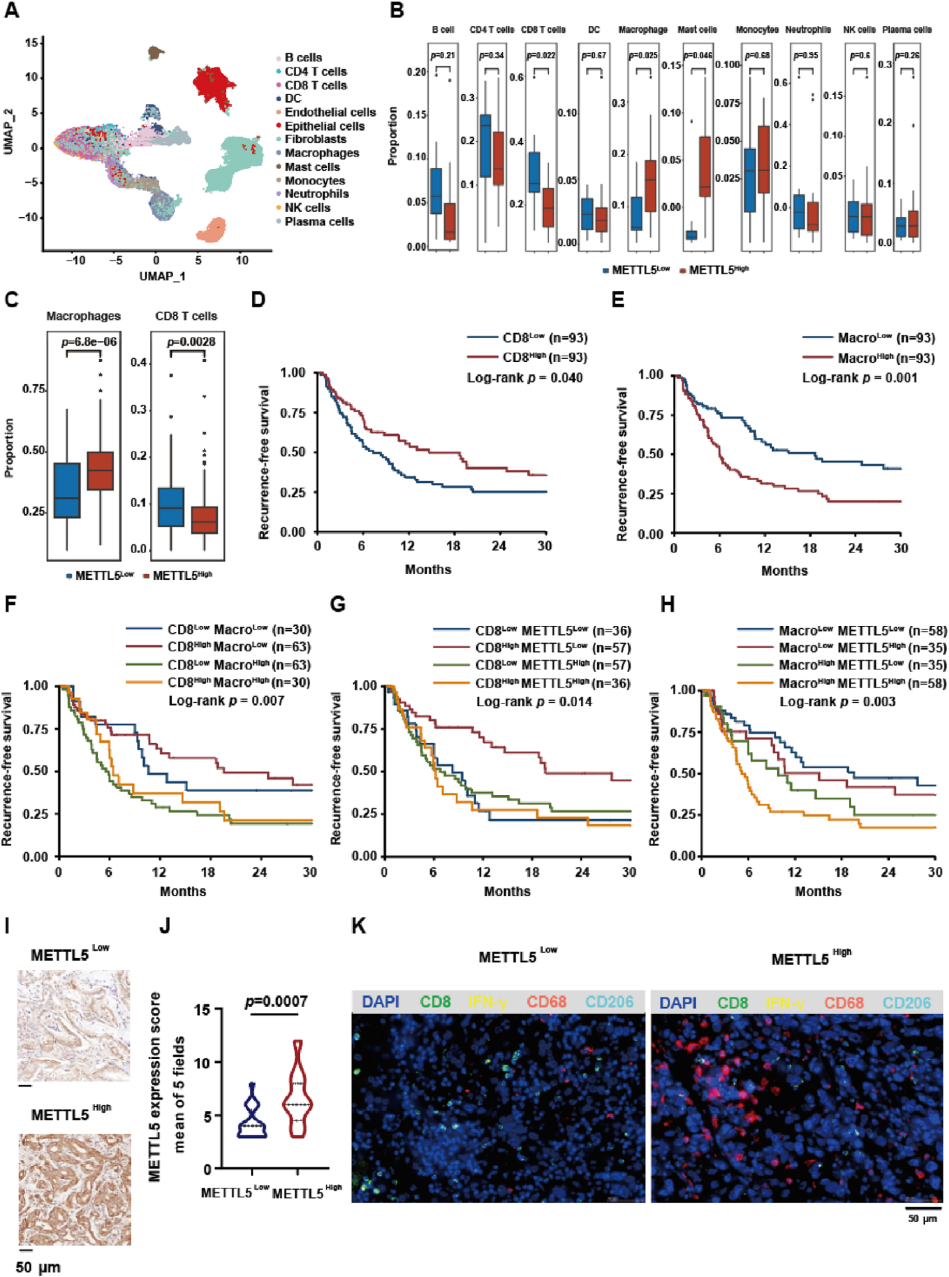
METTL5 expression, macrophage and CD8^+^ T cell infiltration correlates with ICC patient survival. A) scRNA‐seq was performed on 24 human ICC tumors from patients. Based on lineage marker expression, cells were grouped into 13 clusters and illustrated as a UMAP plot. B) Based on the median expression level of METTL5 (0.9319677), patient samples were divided into High and Low group (n=12 vs 12). The proportion and statistical analysis of immune cells including B cells, CD4 T cells, CD8 T cells, DC, endothelial cells, epithelial cells, fibroblast, macrophages, mast cells, monocytes, neutrophils, NK cells, and plasma cells. C) 186 human ICC tumors were used for bulk RNA‐seq. Based on the median expression level of METTL5 (35.8093), patient samples were divided into High and Low group (n=93 vs 93). Immune components including macrophages and CD8^+^ T cells were analyzed using CIBERSORT. D–H) Survival analysis of ICC patients. Patients were divided into different groups based on the median expression of METTL5, proportion of CD8 and macrophage from bulk RNA‐seq data. I,J) IHC staining showing METTL5 expression of ICC samples in High and Low group and statistical analysis (n=20 vs 20). K) mIF staining of CD8, IFN‐γ, CD68, CD206, and DAPI in METTL5 High and Low expression ICC paraffin tissues (n = 20 vs 20).

To validate the role of METTL5 and immune cell infiltration in human ICC, we collected 24 ICC samples for scRNA‐seq (Table S1, Supporting Information). 24 samples were separated into METTL5 high and low expression group based on METTL5 median expression (12 vs 12) (Table S2, Supporting Information). All cells were integrated, partitioned and classified into 13 clusters, including B cells, CD4 T cells, CD8 T cells, dendritic cells (DC), endothelial cells, epithelial cells, fibroblast, macrophages, mast cells, monocytes, neutrophils, NK cells and plasma cells (Figure **3**A; Figure S3A,B, Supporting Information). We then focused on immune cell clusters, and illustrated that macrophages were increased in METTL5^High^ group, accompanied by decreased CD8^+^T cells (Figure 3B). Meanwhile, the cytotoxic score of CD8^+^T cells was significantly elevated in METTL5^Low^ group, while macrophages exhibited higher pro‐tumoral score^[^ ^24^ ^]^ in METTL5^High^ group, indicating an immunosuppressive tumor‐associated macrophage (TAM) phenotype (Figure S3C,D, Supporting Information).

Next, we analyzed a total of 186 ICC bulk RNA‐seq data from our cohort (Table S3, Supporting Information). All ICC samples were separated into METTL5 high and low expression group based on median expression of METTL5 (93 vs 93) (Table S4, Supporting Information). By CIBERSORT analysis, we uncovered that macrophage proportion was increased in METTL5^High^ group, accompanied by reduced CD8^+^ T cell population (Figure 3C). Moreover, high CD8^+^ T cell or low macrophage population alone, or high CD8^+^ T cell with low macrophage population correlated with better ICC recurrence‐free survival, respectively (Figure 3D–F; Figure S3E–G and Tables S5–S7, Supporting Information). We also elucidated that METTL5 low expression with high CD8 infiltration and METTL5 low expression with low macrophage infiltration predicted better recurrence‐free survival in ICC patients (Figure 3G,H; Figure S3H,I and Tables S8 and S9, Supporting Information).

To validate METTL5 expression and immune cell infiltration in these ICC patients, we performed IHC staining and multiplex immunofluorescence (mIF) staining in METTL5 high and low expression samples, and confirmed the relative expression of METTL5 (Figure 3I,J). Of note, the proportion and distribution of CD206^+^CD68^+^ macrophages were significantly increased in METTL5^High^ group, accompanied with decreased IFN‐γ^+^CD8^+^ T cells (Figure 3K; Figure S3J–L, Supporting Information). Taken together, METTL5 correlates with ICC immunosuppressive microenvironment, which predicts patient survival.

### 2.4 TAMs from METTL5 High Expression Group Promote ICC Progression

**Figure 4:**
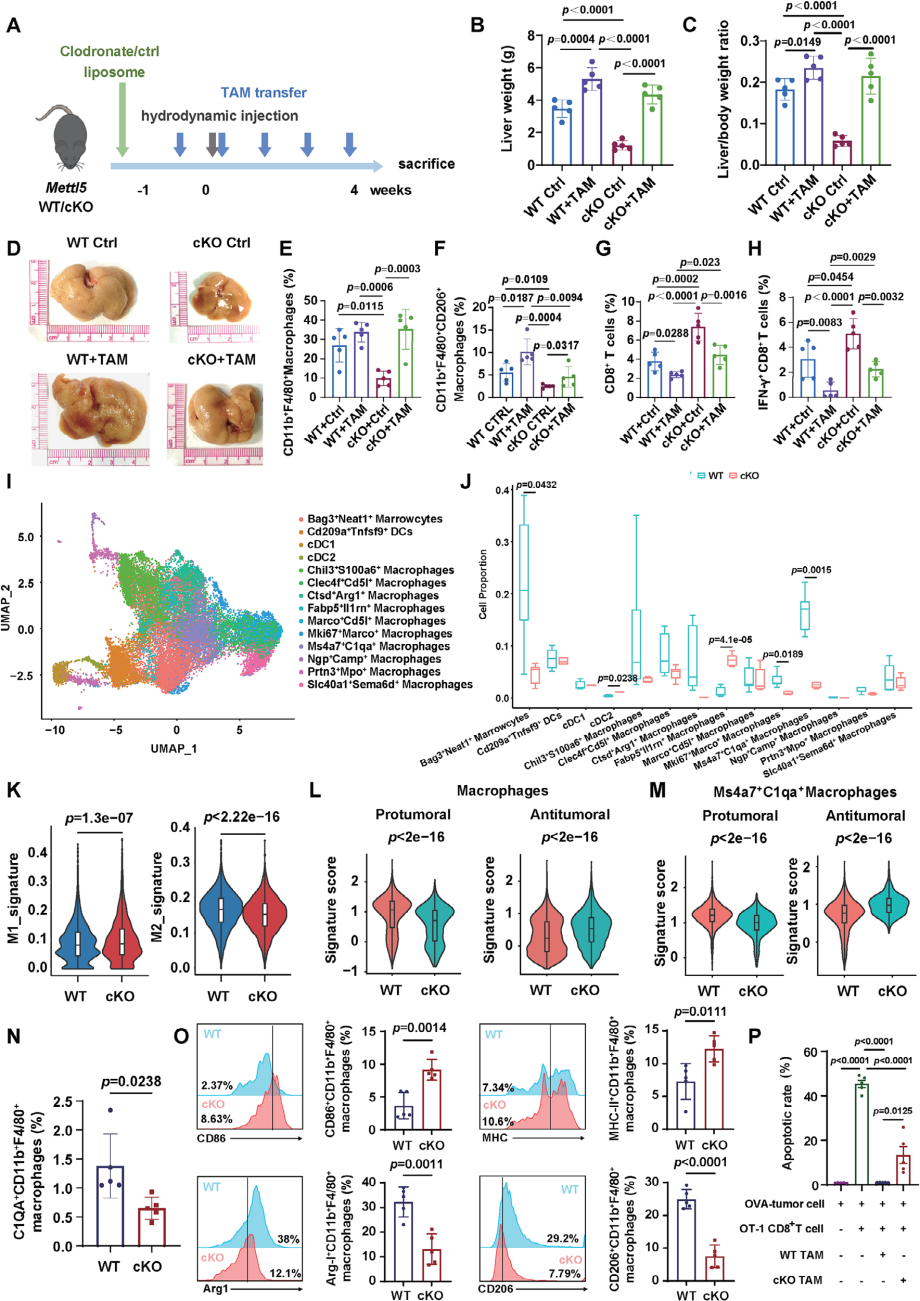
TAM promotes ICC progression in WT and cKO mice. A) Schematic diagram of the experimental design. 6–8 weeks old C57BL/6 mice were administered with Clodronate liposome to deplete macrophages 9 days before ICC establishment by tail vein injection. TAM transfer were performed by tail vein injection once a week for five times. B,C) Liver weight (B) and liver/body weight ratio (C) of the mice (n = 5). D) Representative gross images of mice livers. E–H) Flow cytometry analysis of liver‐infiltrating CD11b^+^F4/80^+^ macrophages, CD11b^+^F4/80^+^CD206^+^ macrophages, CD8^+^ T cells, and IFN‐γ^+^CD8^+^ T cells. I,J) The UMAP plot (I) and statistical analysis (J) of 14 sub‐clusters of myeloid cells. K) Violin plots showing the M1 and M2 signature scores of macrophages in WT and cKO mice. L,M) The pro‐tumoral and anti‐tumoral scores of total macrophages (L) and Ms4a7^+^C1QA^+^ macrophages (M) in WT and cKO mice. N,O) Flow cytometry analysis of liver‐infiltrating C1QA^+^CD11b^+^F4/80^+^ macrophages (N) and expression of CD86, MHC‐II, Arg‐I and CD206 in CD11b^+^F4/80^+^ macrophages (O)(n = 5). P) The percentage of apoptotic OVA‐expressing LTP‐C9 cells alone or co‐cultured with OT‐1 CD8^+^T cells with or without TAMs isolated from WT/cKO mice (n = 5).

Since we observed that TAMs were markedly increased in METTL5 high expression human ICC by scRNA‐seq and bulk RNA‐seq, we next explored the pro‐tumoral functions of TAMs in our ICC model. To this end, mice were hydrodynamically injected with Yap‐S127A, myr‐Akt1 and Sleeping Beauty plasmids to induce ICC establishment as TAM donors. Endogenous macrophages were depleted using Clodronate liposome, followed by ICC establishment. TAMs isolated from WT mice bearing ICC tumors were adoptively transferred to WT and cKO mice once a week (Figure **4**A). Notably, adoptive transfer of TAMs from WT mice significantly promoted ICC progression in WT mice, whereas knockout of *Mettl5* ameliorated ICC progression, which was reverted by additional TAM transfer in cKO mice (Figure 4B–D; Figure S4A,B, Supporting Information). IHC staining (Figure S4C‐H, Supporting Information) and flow cytometry analysis (Figure 4E–H) showed that TAMs were increased in liver after adoptive transfer, accompanied by decreased infiltration of CD8^+^ and IFN‐γ^+^CD8^+^ T cells.

To explore the specific myeloid cell types (Figure 2H,I) that contribute to ICC progression, we further separated myeloid cell cluster into 14 sub‐populations. Interestingly, we found that Ms4a7^+^C1qa^+^ macrophages were remarkably decreased in cKO group, compared to WT group (Figure 4I,J). Previously, Zhang et al. reported that C1QA^+^ macrophages were enriched in human HCC, resembled the signatures for TAMs and associated with poor prognosis.^[^ ^25^ ^]^ To examine the distribution and number of C1qa^+^ macrophages in our mouse model, we performed IHC staining of the mice livers. In consistent with the scRNA‐seq data, both total F4/80^+^ macrophages and C1QA^+^ macrophages were significantly decreased in cKO group (Figure S4I–K, Supporting Information).

Next, we investigated the functions of macrophages and C1qa^+^ macrophages by assessing the pro‐tumoral, anti‐tumoral, M1 and M2 signature scores.^[^ ^24^, ^26^ ^]^ The results showed that macrophages from cKO mice exhibited a lower M2 and pro‐tumoral scores but a higher anti‐tumoral and M1 scores (Figure 4K,L). In addition, the Ms4a7^+^C1qa^+^ macrophages in cKO group also showed less pro‐tumoral phenotype compared to WT group (Figure 4 M). We also performed flow cytometry analysis and validated that C1qa^+^CD11b^+^ macrophages were decreased in cKO mice, with lower expression of CD206 and Arg‐I but higher expression of CD86 and MHC‐II (Figure 4N,O). In addition, we isolated macrophages from WT and cKO mice to co‐culture with CD8^+^ T cells isolated from OT‐1 mice, and detected T cell activation, cytokine release, and direct tumor cell killing. We found that macrophages isolated from WT mice exhibited enhanced immunosuppressive functions compared to those of cKO mice. The expression of T cell activation marker CD69, IFN‐γ, Granzyme B, and tumor cell killing ability of OT1‐CD8^+^ T cells against OVA‐tumor cells were significantly suppressed when co‐cultured with macrophages isolated from WT mice (Figure 4P; Figure S4L–N, Supporting Information). Collectively, we demonstrate that TAMs decrease in *Mettl5* cKO mice and exhibit immunosuppressive phenotype.

### 2.5 Liver‐Specific Knockout of *Mettl5* Unleashes Anti‐Tumor Immunity

**Figure 5:**
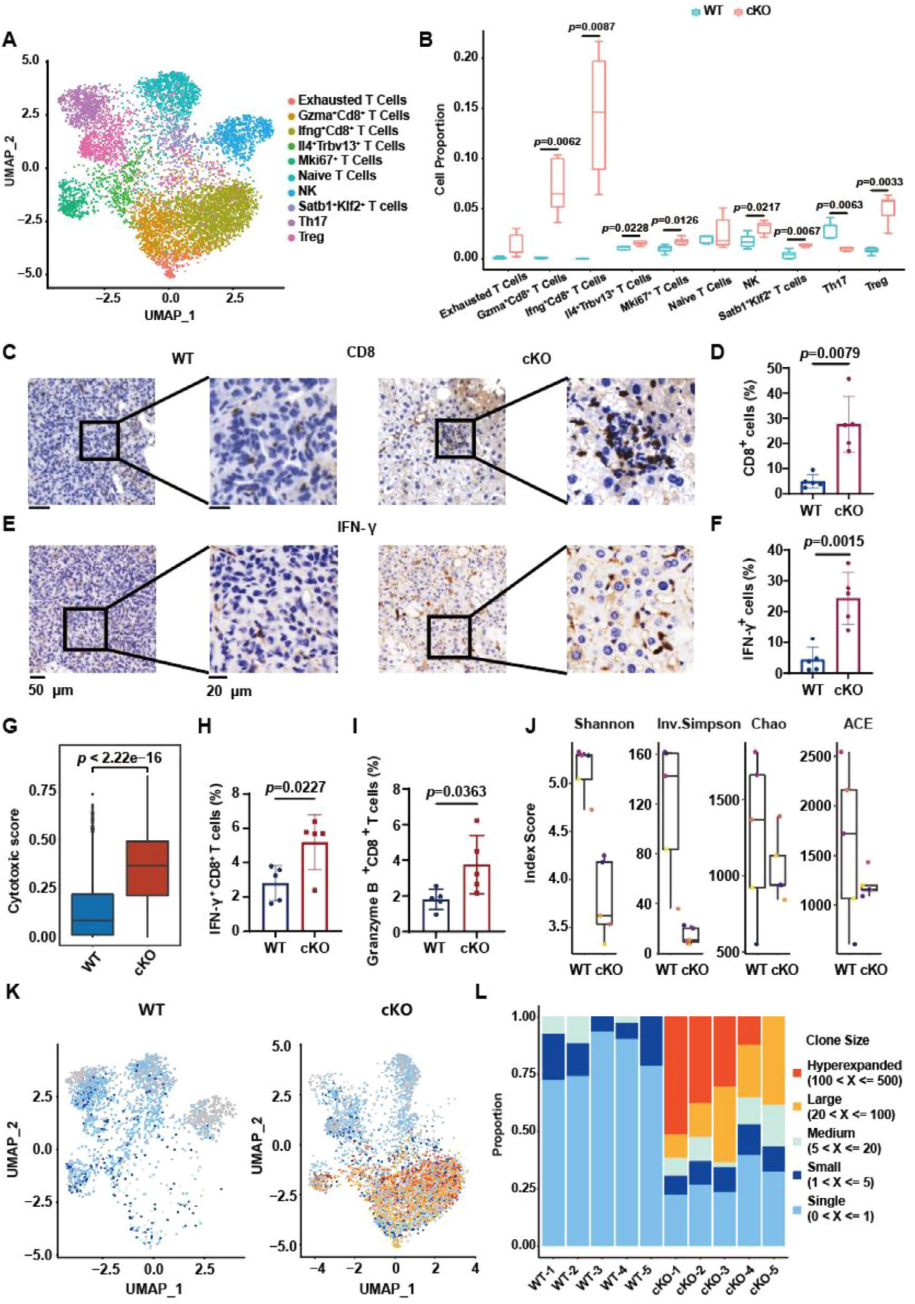
Knockout of *Mettl5* enhances anti‐tumor immunity in liver. A,B) The UMAP plot (A) and statistical analysis (B) of 10 sub‐clusters of T and NK cells. C–F) IHC staining showing CD8/IFN‐γ expression and statistical analysis (n=5). G) Box plots showing the cytotoxic score of CD8^+^ T cells in WT and cKO mice. H,I) Flow cytometry analysis of liver‐infiltrating IFN‐γ^+^CD8^+^ T cells and Granzyme B^+^CD8^+^ T cells (n=5). J) The Shannon, Inv.Simpson, Chao, and ACE index scores that represented TCR diversity in WT and cKO mice. K) The UMAP plot showing the TCR clonal expansion of 10 sub‐populations. L) The proportion of expanded TCR clones in each WT and cKO mice.

Due to the fact that T cells were increased in cKO mouse livers, we next investigated that types of cells were changed by separating T and NK cell cluster into 10 sub‐populations (**Figure** **5**A). Among them, IFN‐γ^+^CD8^+^ effector T cells were remarkably increased in cKO mice compared to those in WT mice (Figure 5B). IHC staining of livers also exhibited increased infiltration of CD8^+^ T cells and IFN‐γ^+^CD8^+^ T cells in cKO mice (Figure 5C–F). In addition, the cytotoxic score^[^ ^27^ ^]^ of CD8^+^ T cells was significantly higher in cKO group, suggesting enhanced cytotoxic functions and anti‐tumor immunity upon *Mettl5*‐depletion (Figure 5G). We further confirmed the increased proportion of IFN‐γ^+^CD8^+^ T cells and Granzyme B^+^CD8^+^ T cells by flow cytometry analysis (Figure 5H,I).

Furthermore, we also performed single cell T cell receptor (TCR) sequencing and elucidated that the Shannon, Inv.Simpson, Chao and ACE index scores that represented TCR diversity were markedly decreased in cKO group compared to WT group (Figure 5J). We then compared the clone size of the TCR clonotypes and uncovered that the CD8^+^ effector T cell cluster but not other sub‐populations were predominantly hyperexpanded in cKO group, while most clonotypes were less expanded in WT group (Figure 5K,L). Furthermore, the clonal expansion of CD39^+^CD103^+^ T cells and T cells that has been reported as tumor specific T cells^[^ ^28^, ^29^ ^]^ were also increased in cKO group, indicating that the anti‐tumor immunity was enhanced in cKO mice (Figure S5A,B, Supporting Information). However, BCR diversity and clonal expansion were comparable between WT and cKO group via single cell B cell receptor (BCR) sequencing analysis (Figure S5C–E, Supporting Information). These findings unveil that knockout of *Mettl5* promotes effector T cell infiltration and expansion to suppress ICC progression.

### 2.6 METTL5 Translationally Regulates Chemokine Expression

**Figure 6:**
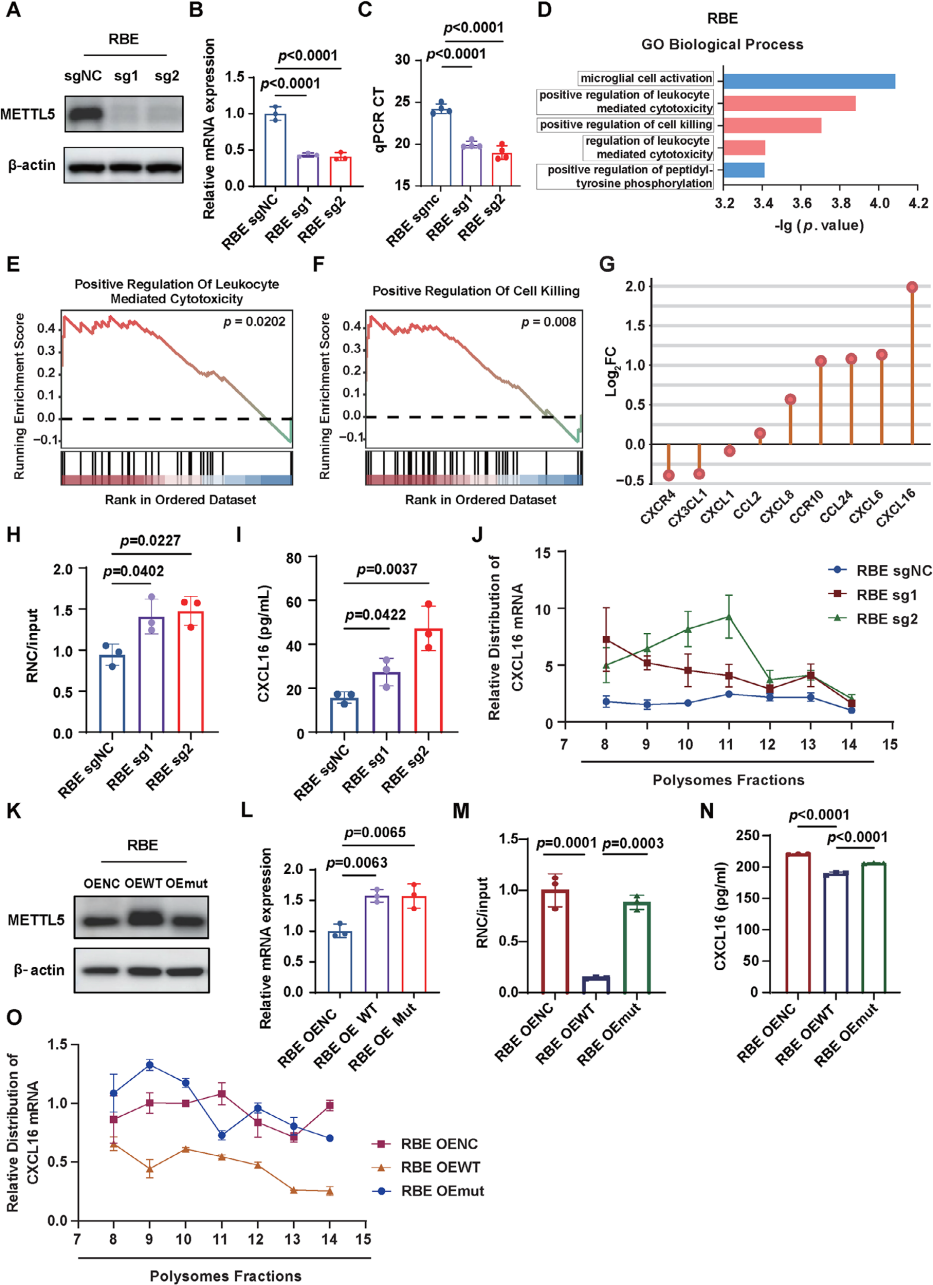
METTL5 regulates the mRNA translation of *CXCL16*. A,B) Western blot and QPCR analysis of METTL5 in RBE sg*NC* and sg*METTL5* (sg1, sg2) cells. C) SELECT analysis showing the m^6^A_1832_ modification of 18S rRNA in sg*NC*, sg1, and sg2 cells. D) Top five enriched pathways of translationally upregulated genes by GO analysis (Biological Process). E,F) Gene set enrichment analysis (GSEA) validated the enrichment of positive regulation of leukocyte mediated cytotoxicity and positive regulation of cell killing pathways. G) The differential translated chemokines and receptors comparing RBE sg*NC* and sg*METTL5* cells. H–J) RNC‐qPCR (H), ELISA (I), and polysome profiling (J) showing the *CXCL16* mRNA translation ratio, protein level and polysome fractions in RBE sg*NC* and two sg*METTL5* cells. In panel H, the expression of *CXCL16* in RNC pellet was divided by the total mRNA expression of *CXCL16* (input), and this value indicated the *CXCL16* mRNA translation ratio. K,L) Western blot and QPCR analysis of METTL5 in RBE OE NC, OE WT, and OE Mut cells. M–O) RNC‐qPCR (M), ELISA (N), and polysome profiling (O) showing the *CXCL16* mRNA translation ratio, protein level, and polysome fractions in RBE OE NC, OE WT, and OE Mut cells. (n = 3).

To investigate the mechanisms underlying METTL5‐mediated immune microenvironment reshaping, we established *METTL5* knockout (sg1, sg2) in RBE cells using CRISPR‐Cas9 system (Figure **6**A,B). The m^6^A_1832_ modification of 18S rRNA was significantly decreased in *METTL5* knockout cells, suggesting the successful *METTL5* knockout (Figure 6C). Since METTL5‐mediated ribosome 18S rRNA m^6^A_1832_ modification regulated mRNA translation, we performed Ribosome nascent‐chain complex‐bound mRNA sequencing (RNC‐seq) and ribosome profiling (Ribo‐seq) on RBE control and *METTL5* knockout cells. By GO and GSEA, we found that pathways related to positive regulation of leukocyte mediated cytotoxicity were enriched upon *METTL5* knockout (Figure 6D–F). As we observed that METTL5 modulated immune cell infiltration in the liver, we next explore chemokines that were translationally regulated by METTL5. The results showed that CXCL16, which has been recognized as an important chemokine recruiting cytotoxic T lymphocytes,^[^ ^30^ ^]^ was notably upregulated in RBE sg*METTL5* cells compared to control cells (Figure 6G). To validate this finding, we also constructed control and *Mettl5* knockout (sg1, sg2) in mouse ICC cells LTP‐C9,^[^ ^23^ ^]^ and performed RNC‐qPCR, ELISA and polysome‐qPCR analysis in both RBE and LTP‐C9 cells. We demonstrated that knockout of *METTL5* markedly increased *CXCL16* mRNA translation and protein levels (Figure 6H–J; Figure S6A–E, Supporting Information). Consistently, increased CXCL16 expression was also observed in METTL5^Low^ ICCs (Figure S6F,G, Supporting Information). Furthermore, we constructed a mutation of METTL5 putative catalytic motif NPP to AAA (amino acids 127‐129, abbreviated METTL5‐Mut)^[^ ^31^ ^]^ and transfected RBE cells with control or METTL5 wildtype (WT) or mutant (Mut) plasmids (Figure 6K,L), and performed RNC‐QPCR, ELISA, and polysome‐qPCR analysis to detect CXCL16 expression level in these cells. Intriguingly, overexpression of *METTL5*‐WT significantly decreased *CXCL16* mRNA translation and protein levels, while there was no difference between *METTL5*‐Mut and control groups (Figure 6M–O), indicating that METTL5 methylase activity is necessary for regulating *CXCL16* mRNA translation.

In addition, we also found that *CX3CL1* was translationally downregulated in sg*METTL5* cells compared to sg*NC* cells (Figure 6G). CX3CL1 is a unique CX3C chemokine, which recruits TAMs through binding to its receptor CX3CR1 expressed on monocytes/macrophages.^[^ ^32^ ^]^ Similarly, METTL5 depletion markedly hampered CX3CL1 mRNA translation and protein level (Figure S7A–F, Supporting Information). Decreased CX3CL1 expression was also observed in human METTL5^Low^ ICCs (Figure S7G,H, Supporting Information). scRNAseq analysis of WT and cKO mouse livers showed that CX3CR1, the receptor of CX3CL1, was predominantly expressed by myeloid cells and macrophage clusters, which were increased in WT mice (Figure S7I, Supporting Information). These data suggests that METTL5 mediated *CXCL16* and *CX3CL1* mRNA translation and expression to reshape liver immune microenvironment and promote ICC progression.

### 2.7 METTL5‐Mediated CXCL16 Downregulation Suppresses the Migration of Cytotoxic T Cells

**Figure 7:**
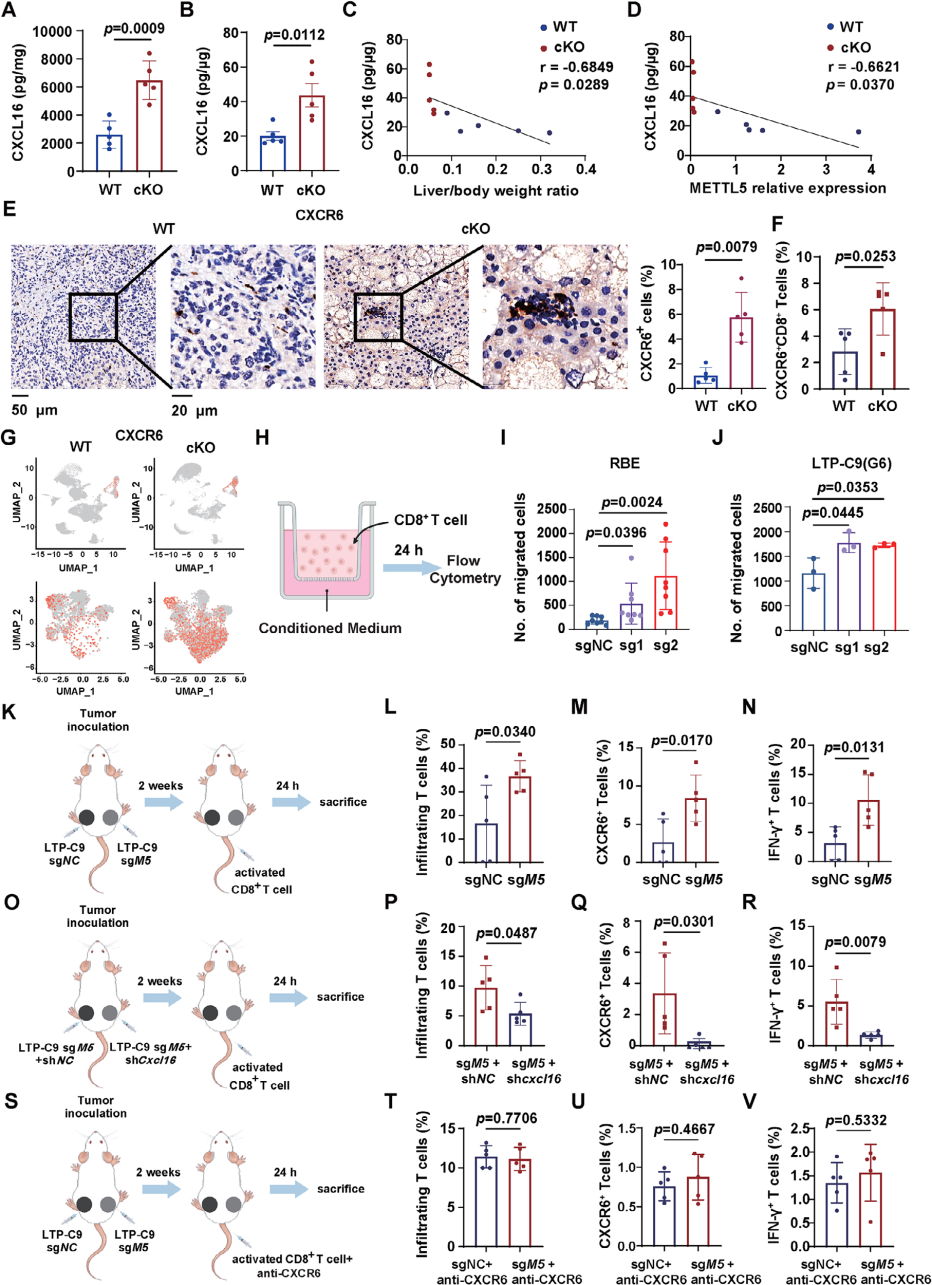
METTL5 suppresses CXCL16 expression to reduced cytotoxic T cell chemotaxis. A,B) The protein level of CXCL16 in WT and cKO mice livers detected by ELISA, and normalized to liver weight (A) and total protein level (B), respectively. C,D) The correlation between CXCL16 level and liver to body weight ratio (C) or METTL5 expression (D). E) IHC staining and statistical analysis of CXCR6 expression in WT and cKO mice livers. F) Flow cytometry analysis of CXCR6^+^CD8^+^ T cells. (G) The UMAP plot showing CXCR6 positive cells in all clusters (upper panel) and T and NK cell clusters (lower panel). (H) Schematic diagram of in vitro T cell migration experiment. I,J) Number of migrated CD8^+^ T cells in RBE or LTP‐C9(G6) sg*NC*/sg*Mettl5* cell conditioned medium. K) Schematic diagram of in vivo T cell migration experiment. NCG mice were injected with LTP‐C9 sg*NC* and sg*Mettl5* cells in the left and right flank, respectively, followed by activated CD8^+^ T cell transfer (n = 5). (L–N) Flow cytometry analysis of infiltrating CD8^+^ T cells, CD8^+^CXCR6^+^ T cells and IFN‐γ^+^CD8^+^CXCR6^+^ T cells. O) Schematic diagram of in vivo T cell migration experiment. NCG mice were injected with LTP‐C9 sg*Mettl5* + sh*NC* cells and LTP‐C9 sg*Mettl5* + sh*Cxcl16* cells in the left and right flank, respectively, followed by activated CD8^+^ T cell transfer (n=5). P–R) Flow cytometry analysis of infiltrating CD8^+^ T cells, CD8^+^CXCR6^+^ T cells, and IFN‐γ^+^CD8^+^CXCR6^+^ T cells (n=5). S) Schematic diagram of in vivo T cell migration experiment. NCG mice were injected with LTP‐C9 sg*NC* and sg*Mettl5* cells in the left and right flank, respectively, followed by activated CD8^+^ T cell transfer incubated with CXCR6 antibody (n=5). T–V) Flow cytometry analysis of infiltrating CD8^+^ T cells, CD8^+^CXCR6^+^ T cells, and IFN‐γ^+^CD8^+^CXCR6^+^ T cells (n=5).

Next, we detected CXCL16 expression in WT and cKO mouse livers by ELISA, and found that CXCL16 protein levels were remarkably increased in cKO group (Figure **7**A,B). In addition, CXCL16 expression was negatively correlated with liver/body weight ratio representing tumor burden and METTL5 expression (Figure 7C,D). IHC staining of CXCR6, the receptor of CXCL16, also showed increase of CXCR6^+^ cells in cKO mouse livers (Figure 7E), which was validated by flow cytometry (Figure 7F). Of note, CXCR6 was predominantly expressed in lymphoid cells and increased in effector T cells of cKO mice analyzed by scRNA‐seq (Figure 7G).

To verify knockout of *METTL5/Mettl5* in RBE/LTP‐C9 cells promotes CXCL16 expression to increase CD8^+^ T cell chemotaxis in vitro, we seeded RBE/LTP‐C9 sgNC/sg*METTL5/Mettl5* cells in the lower chamber of transwell, respectively, and added CD8^+^ T cells on the upper chamber. After 24 h, CD8^+^ T cells migrated to the lower chamber were collected for counting. Intriguingly, the numbers of migrated CD8^+^ T cells were increased in both RBE sg*METTL5* and LTP‐C9 sg*Mettl5* group compared to sg*NC* groups (Figure 7H–J). Next, we further investigate whether METTL5‐mediated CXCL16 downregulation modulated CD8^+^ T cell migration in vivo. We injected LTP‐C9 sg*NC* and sg*Mettl5* cells on the left and right flank of NCG mice, respectively. Two weeks later, activated CD8^+^ T cells were adoptively transferred to the mice by tail vein injection. All mice were sacrificed 24 h later for tumor collection and flow cytometry analysis (Figure 7K). Interestingly, the infiltrating CD8^+^ T cells, CXCR6^+^CD8^+^ T cells and IFN‐γ^+^CD8^+^ T cells were all increased in the flank of sg*Mettl5* tumors (Figure 7L–N). We further established *Cxcl16* knockdown on LTP‐C9 sg*Mettl5* cells, and injected sg*Mettl5*+sh*NC* and sg*Mettl5*+sh*Cxcl16* LTP‐C9 cells on different flank of NCG mice, respectively (Figure 7O). Knockdown of *Cxcl16* was confirmed by QPCR and ELISA (Figure S8A,B, Supporting Information). Interestingly, the additional depletion of *Cxcl16* profoundly impaired the tumor infiltration of according T cells (Figure 7P–R). Furthermore, CXCR6 blockade abrogated the recruitment of CD8^+^ T cells, CXCR6^+^CD8^+^ T cells, and IFN‐γ^+^CD8^+^ T cells mediated by *Mettl5* depletion (Figure 7S–V), indicating that CXCR6 is responsible for CD8^+^ T cell recruitment. Taken together, these findings indicate that METTL5 suppresses tumor infiltration of CD8^+^ T cells via translational modulation of *CXCL16* expression.

### 2.8 Knockdown of *Mettl5* Enhanced Response to Anti‐PD‐1 Immunotherapy in Mice

CD8^+^ T cell infiltration and immunosuppressive microenvironment have been demonstrated as important indicators of response to immunotherapies. Due to the potent role of METTL5 in shaping ICC immune microenvironment, we sought to investigate the expression of immune checkpoint molecules including PD‐1 in WT and cKO mice and ICC patient samples by scRNA‐seq analysis. Interestingly, the expression of PD‐1 on CD8^+^ T cell and cytotoxic effector molecules was markedly enhanced in *METTL5* low expression ICC patients (Figure S9A–C, Supporting Information) and cKO mice (Figure S9D–F, Supporting Information). In addition, we also found that the proportion and distribution of CD68^+^CD206^+^C1QA^+^ macrophages were significantly decreased in METTL5^Low^ group, accompanied with increased PD‐1^+^IFN‐γ^+^CD8^+^ T cells (Figure S9G–I, Supporting Information).

**Figure 8:**
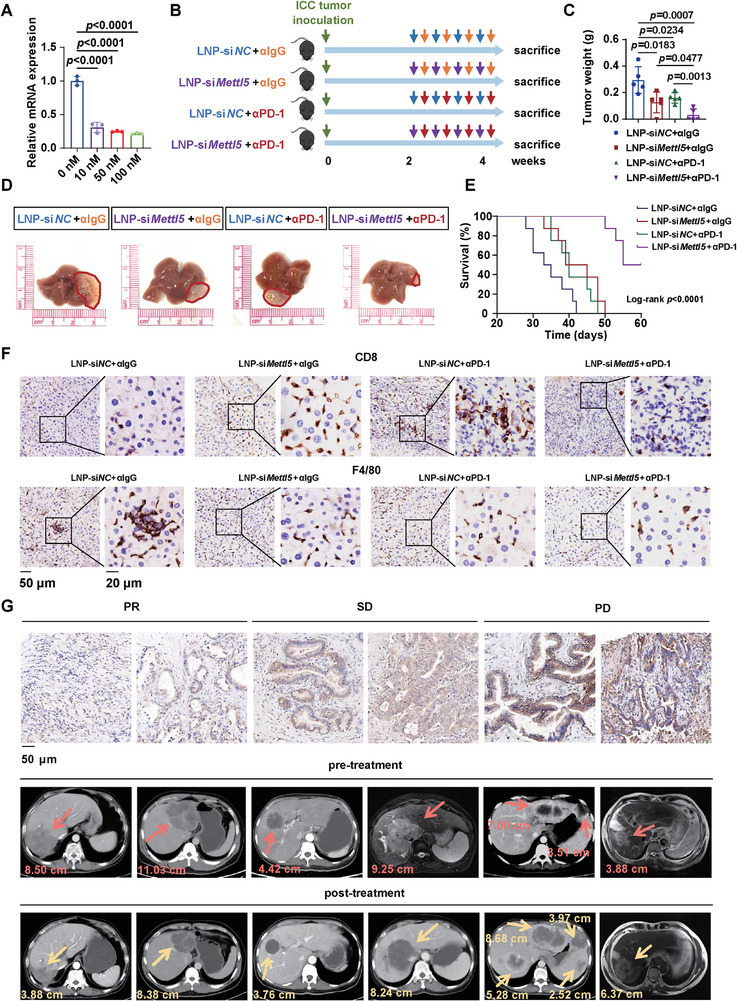
The combination of *Mettl5* knockdown and anti‐PD‐1 treatment prominently suppresses ICC tumors in mice. A) QPCR analysis of *Mettl5* expression to validate the knockdown efficiency of LNP‐si*Mettl5* in LTP‐C9 cells. B) Schematic diagram of experimental design. All mice were intrahepatically injected with LTP‐C9 cells, and administered with 4 doses of LNP‐si*NC/Mettl5* and αIgG/αPD‐1 every 3 days, respectively (n=5). C) Tumor weight of the mice. D–F) Representative gross images (D), survival (E), and IHC staining of CD8 and F4/80 (F) of the mice livers. G) IHC staining of METTL5 and images showing ICC tumor size before and after treatment of ICC patients who have received immunotherapy. PR, partial response, n = 3; SD, stable disease, n = 4; PD, progression disease, n = 10.

Next, we investigated the efficacy of combined *METTL5* knockdown and PD‐1 blockade in treating ICC. As there are no specific small‐molecule inhibitors for METTL5, we knockdown *Mettl5* in vivo by gene silencing using small interfering RNA (siRNA). We demonstrated that *Mettl5* siRNA lipid nanoparticle (LNP) could be successfully uptaken by LTP‐C9 cells and displayed potent knockdown efficiency (Figure **8**A). Furthermore, we orthotopically injected LTP‐C9 cells into C57BL/6 mice, and administered these mice with LNP‐si*NC/Mettl5* and αIgG/αPD‐1, respectively (Figure 8B). The results showed that single αPD‐1 or LNP‐si*Mettl5* treatment significantly suppressed ICC tumor progression, while the combination of αPD‐1 and LNP‐si*Mettl5* profoundly eliminated ICC tumors and prolonged survival (Figure 8C–E). Since ICC tumors of many mice in combined treatment group were eradicated, we examined the T cell and macrophage infiltration and distribution by IHC staining, and observed the elevated CD8^+^ T cells and decreased macrophages in livers after treatment of αPD‐1 or/and LNP‐si*Mettl5* (Figure 8F; Figure S10A,B, Supporting Information).

To access whether METTL5 expression could predict response to immunotherapy, we collected biopsy samples from 17 ICC patients (three patients with partial response, PR; four patients with stable disease, SD; ten patients with progression disease, PD) before immunotherapy to detect METTL5 expression by IHC staining and followed up treatment responses. We found that patients with higher METTL5 expression were more likely to resist to immunotherapy (Figure 8G; Figure S10C, Supporting Information), which awaits further validation by a larger patient cohort in the future.

## 3 Discussion

The liver immune microenvironment is essential to cancer development, progression, and treatment response. In this study, we reveal that *Mettl5* shapes the immunosuppressive liver microenvironment that facilitates ICC progression. By scRNA‐seq analysis of mouse and human ICC, we demonstrate that METTL5 creates a TAM‐enriched and T cell‐excluded and ‐dysfunctional microenvironment, which correlated with patient survival. Mechanistically, METTL5 modulates the *CXCL16* mRNA translation to exclude CD8^+^ T cell infiltration, thus enabling immune evasion. The combination of *Mettl5* targeting and PD‐1 blockade prominently suppresses ICC progression. In addition, high *METTL5* expression correlated with poor response to PD‐1 blockade in ICC patients. Our study suggests METTL5 as a promising targeting for ICC intervention via synergizing with immunotherapy.

METTL5, an enzyme responsible for catalyzing 18S rRNA m^6^A_1832_ modification, is essential for efficient translation. Our data from tumor‐free *Mettl5*‐cKO mice provide critical in vivo evidence for a favorable therapeutic window. *Mettl5*‐cKO mice developed normally and exhibited no overt signs of systemic toxicity or gross physiological abnormalities during our observation period. This suggests that normal tissues possess a remarkable tolerance for the loss of m^6^A_1832_ modification, potentially due to compensatory mechanisms or a lower dependency on hyper‐efficient translation compared to rapidly proliferating cancer cells. This phenomenon aligns with the targeting of other essential ribosomal proteins or modifiers in cancer therapy, where a differential dependency between malignant and normal cells creates a therapeutic opportunity. Thus, while comprehensive toxicology studies are imperative for clinical translation, our findings suggest that METTL5 inhibition represents a viable and promising strategy with a manageable safety profile.

In the recent years, accumulating studies have highlighted the novel role of epitranscriptome in regulating cancer development and tumor microenvironment (TME). Li et al. reported that the m^6^A/m^5^C/m^1^A signature genes significantly correlated with liver cancer tumor stage, prognosis and immune cell infiltration.^[^ ^33^ ^]^ Of note, multiple RNA modification related genes including “writers”, “readers” and “erasers”, have been reported to be involved in liver cancer development by promoting lipogenesis,^[^ ^34^ ^]^ regulating PD‐L1 expression on tumor cells,^[^ ^35^, ^36^ ^]^ and promoting PD‐L1^+^ macrophage recruitment^[^ ^37^ ^]^ through the regulation of mRNA and tRNA modification. In this study, for the first time we demonstrate that METTL5‐mediated ribosomal RNA m^6^A modification shapes the liver immune microenvironment, especially excludes T cell. T cell infiltration into tumors is pivotal to tumor control and clinical outcomes. Mechanisms that coordinate T cell exclusion are incompletely understood. We have demonstrated that METTL5 downregulates the translation of *CXCL16* mRNA to exclude IFN‐γ^+^CD8^+^ T cells, and thus promoting ICC progression. This data defines a fundamental link between liver and tumor immunobiology wherein hepatocytes govern productive T cell surveillance in liver cancer, and provides the novel evidence for the indispensable role of posttranscriptional regulation in reshaping immune microenvironment. As expected, the potential readers and erasers for these ribosomal RNA modifications may also play important roles in remodeling ICC immune microenvironment. Therefore, identification and validation of these key regulators are desired for further studies.

The immune microenvironment supports ICC growth and progression through various mechanisms, including induction of angiogenesis, immunosuppression, and enhanced tumor cell migration.^[^ ^38^, ^39^, ^40^ ^]^ TAMs are a population of plastic immune cells that are activated and infiltrated at tumor sites, possessing potent pro‐tumoral and immunosuppressive function. It has been reported that increased TAM infiltration correlated with ICC poor survival,^[^ ^41^ ^]^ predominantly exhibiting as M2 phenotype.^[^ ^42^ ^]^ Here, we reveal that *Mettl5* depletion significantly reduces tumor‐infiltrating Ms4a7^+^C1qa^+^ macrophages in ICC. Adoptive transfer of TAMs from ICC tumors of WT mice significantly promoted ICC progression in both WT and cKO mice, indicating the pro‐tumoral function of TAMs. Complement C1q has been identified as a marker of a tolerogenic and immunosuppressive macrophage populations in both healthy and tumor tissues. The presence of C1q^+^ macrophages correlated with T cell exhaustion and poor prognosis in many cancers, but its role remained poorly understood.^[^ ^43^ ^]^ Previously, Zhang et al. also showed that C1QA macrophages possessed TAM‐like functions, and associated with poor clinical outcomes in HCC.^[^ ^25^ ^]^ Similarly, we found that TAMs correlated with poor prognosis in our ICC cohort. And these macrophages potently suppressed the proliferation of CD8^+^ T cells, consistent with previous findings.^[^ ^21^ ^]^ However, whether and how METTL5 regulates other cells, such as fibroblasts, endothelial cells to further reshaping ICC microenvironment, and the potential mechanisms underlying METTL5‐ribosomal m^6^A modification‐mediated preferential translation of specific genes are worth further investigation.

In the past decade, immune checkpoint blockades (ICBs) have revolutionized the management of cancers.^[^ ^44^ ^]^ However, the efficacy of ICBs is less than 20% in liver cancer, including ICC. The combination of immunotherapy and chemotherapy has been used as the standard‐of‐care systemic frontline treatment in cholangiocarcinoma patients. And multiple immune‐based therapies are under investigation,^[^ ^40^ ^]^ including Durvalumab plus gemcitabine and cisplatin.^[^ ^45^ ^]^ In our study, we have elucidated that METTL5 was a promising target in combination with PD‐1 blockade for treating ICC. However, there is no specific METTL5 inhibitor available. Alternatively, we employed siRNA‐based therapies, which have entered the pharmaceutical market, with several siRNA‐based drugs approved for clinical use, and several candidates being evaluated in Phase 3 clinical trials.^[^ ^46^, ^47^ ^]^ Since the efficacy of in vivo gene silencing by LNP‐siRNA has been well established,^[^ ^47^, ^48^ ^]^ we knockdown *Mettl5* with this approach to reprogram the immune microenvironment, and combined anti‐PD‐1 therapy to treat ICC. We found that the combination therapy prominently inhibited ICC progression, providing a potential therapeutic strategy for combating ICC.

## 4 Conclusion

In sum, we reveal the pivotal role of METTL5 in reshaping the immune microenvironment by translational regulation of chemokine mRNA, highlighting the potential strategy of targeting METTL5 in combination with anti‐PD‐1 for treating ICC.

## 5 Experimental Section

### Human Specimens

Twenty four fresh ICC tumor specimens were collected for scRNA‐seq analysis and 186 ICC tumors were used for bulk RNA‐seq, and these samples were also used for IHC staining and mIF staining. 17 ICC biopsy samples from patients who have received immunotherapy were used for IHC staining. All samples were collected with patient written consent at the First Affiliated Hospital of Sun Yat‐sen University, Guangzhou, China, from 2012 to 2021. This study was conducted according to the principles of the Declaration of Helsinki and was approved by the Institutional Review Board of The First Affiliated Hospital of Sun Yat‐sen University (Approval No. [2023]766).

### Statistical analysis

Data are presented as mean ± SEM from at least three independent experiments. Comparisons between two groups were performed using the independent Student's t test or Wilcoxon's test, and one‐way ANOVA was used to compare data in more than two groups by GraphPad Prism 8.3.0 (GraphPad Software). Kaplan‐Meier survival analysis was conducted, with significance assessed using the log‐rank test. *P* values are reported in the figures where appropriate. A two‐tailed *p* value of < 0.05 was regarded as statistically significant.

Comprehensive information on methods is provided in Supporting Information.

## Conflict of Interest

The authors declare no conflict of interest.

## Author Contributions

S.L., X.Z., T.S., and Q.C. contributed equally to this work. X.Z.Z., Z.D., S.P., SB.L., J.G., and M.K. performed conceptualization; X.Z.Z., Z.D., X.Z., S.L., and T.S. performed data curation; X.Z.Z., X.Z., S.L., T.S., and J.C. performed formal Analysis; X.Z.Z., Z.D., SB.L., and M.K. performed Funding acquisition; X.Z.Z., Z.D., X.Z., S.L., T.S., Y.W., and Y.Z. performed investigation; XZ.Z., X.Z., S.L., T.S., Y.W., and Y.Z. performed methodology; X.Z.Z., S.P., SB.L., J.G., and M.K. performed project administration; S.B.L., S.P., and M.K. acquired resources; X.Z.Z., X.Z., S.L., and T.S. acquired software; S.P., J.G., Z.P., SB.L., and M.K. performed supervision; XZ.Z., X.Z., S.L., and T.S. performed validation; XZ.Z., X.Z., S.L., and T.S. performed visualization; XZ.Z., X.Z., S.L., and T.S. wrote the original draft; XZ.Z., S.P., A.S.L.C., J.G., Z.P., SB.L., and M.K. reviewed and edited the final manuscript; All authors read and approved the manuscript.

## Supporting information



Supporting Information

## Data Availability

The RNC‐seq and Ribo‐seq data are available in the Genome Sequence Archive located at http://bigd.big.ac.cn/gsa‐human with the accession numbers HRA006821. The scRNA‐seq and bulk RNA‐seq data have been deposited in the OMIX, China National Center for Bioinformation/Beijing Institute of Genomics, Chinese Academy of Sciences (https://ngdc.cncb.ac.cn/omix; Accession number: OMIX012123, OMIX012124). All other data supporting the findings of this study are available from the corresponding author on reasonable request. Source data are provided with this paper.
