# Supplementary material for: METTL5 Enables Immune Evasion of Liver Cancer via Chemokine mRNA Translation Regulation
Source: Adv Sci (Weinh). 2025 Dec 23;13(11):e12528. doi: 10.1002/advs.202512528 (PMC12931177; doi:10.1002/advs.202512528)
Supplement: Supplementary file 1 — Supporting Information [file ADVS-13-e12528-s001.docx]

**METTL5 ENABLES IMMUNE EVASION OF LIVER CANCER VIA CHEMOKINE mRNA TRANSLATION REGULATION**

Shuang Li^1,*^, Xiao Zhao^2,*^, Tongtong Song^1,*^, Qiaoyi Chen^3,*^, Yanqing Wu^4^, Yuting Zhang^5^, Jingying Chen^1^, Yifan Wu^4^, Bin Li^6^, Xinyue Zhang^3^, Zihao Dai^1^, Lixia Xu^3^, Yubin Xie^7^, Alfred Sze-Lok Cheng^8^, Jianping Guo^7^, Ming Kuang^1,7^,Shuibin Lin^9,†^, Zhenwei Peng^2,†^, Sui Peng^4,6,7,†^, Xuezhen Zeng^1,7,†^

**Table of Contents**

Supplementary Materials and Methods 3

Supplementary Figure 1 22

Supplementary Figure 2 23

Supplementary Figure 3 25

Supplementary Figure 4 27

Supplementary Figure 5 29

Supplementary Figure 6 30

Supplementary Figure 7 31

Supplementary Figure 8 33

Supplementary Figure 9 34

Supplementary Figure 10 36

Supplementary Table 1 37

Supplementary Table 2 37

Supplementary Table 3 38

Supplementary Table 4 38

Supplementary Table 5 41

Supplementary Table 6 41

Supplementary Table 7 42

Supplementary Table 8 42

Supplementary Table 9 43

Supplementary Table 10 44

Supplementary Table 11 45

# Supplementary Materials and Methods

**Cell line construction**

12 μg lentiviral vectors expressing sgRNA against *METTL5/Mettl5*, sh*Cxcl16*, the full - length open reading frame (ORF) of the OVA gene or control sequence (sg*NC*/sh*NC*) were co-transfected with 8 μg pCMVDR8.9 and 4 μg pCMV-VSVG into 293T cells using Lipofectamine 3000 reagent (Invitrogen) and Opti-MEM (Thermo Fisher) for lentivirus production. Viruses were collected from cell culture supernatant, and were used to infect RBE (National Collection of Authenticated Cell Cultures, SCSP-557) or LTP-C9 cells ^1^ with 8 μg/ml Polybrene (Solarbio). Puromycin (4 μg/ml) and G418 (1500 μg/ml) were used to select the successfully infected cells for 2 weeks. Knockdown or knockout or overexpression efficiency were validated by QPCR and Western blot analysis.

**Cell counting kit-8 (CCK8) assays**

LTP-C9-G6 sg*NC* or sg*Mettl5* cells were digested and subsequently diluted to a concentration of 10 cells/µl using DMEM medium supplemented with 10% FBS. A volume of 100 μL (1,000 cells/well) of the cell suspension was seeded into 96-well plates in triplicate. The plates were then incubated in a humidified incubator at 37°C, 5% CO_2_ for 6, 24, 48, 72, and 96 hours. At each time point, 10 µL of CCK-8 solution (Dojindo, Japan) was added to each well, followed by 2-hour incubation. The OD value representing absorbance at 450 nm was measured using a microplate reader (Thermo, USA).

**In vitro siRNA transfection**

Mouse negative control siRNA (si*NC,* sense 5’-3’: UUCUCCGAACGUGUCACGUdTdT; antisense 5’-3’: ACGUGACACGUUCGGAGAAdTdT) and three si*Mettl5* with 2’-O-Methyl (2’-OMe) modification were synthesized by Guangzhou IGE Biotechnology. 5 μl si*NC* and si*Mettl5* at final concentrations of 0 nM, 10 nM, 50 nM, and 100 nM were used to transfected LTP-C9 cells using 5 μl Lipofectamine 3000 reagent and 250 μl Opti-MEM. After 48 hours of transfection, cells were harvested to assess knockdown efficiency by RT-qPCR *in vitro*. The si*Mettl5* (sense 5’-3’: GCCCAAGUUACUUCUAGAAdTdT; antisense 5’-3’: UUCUAGAAGUAACUUGGGCTdT) sequence with highest knockdown efficiency was chosen for *in vivo* knockdown.

**Mouse strains, tumor models and treatments**

*Mettl5*^fl/fl^ mice, generated as previously described ^2^, were crossed with Alb-Cre mice to generate liver-specific conditional *Mettl5* knockout mice (cKO). Cre^-/-^ littermates were used as wild-type (WT) mice. NCG mice (6-8 weeks) and wild-type C57BL/6 (4-6 weeks) were purchased from the GemPharmatech. All mice were bred and maintained under specific pathogen-free, 12 h light/dark cycle, temperature of ~18-23℃, 40-60% air humidity conditions in the animal facility of the Animal Experiment Center of Sun Yat-sen University. All studies were approved and supervised by the Animal Care and Use Ethics Committee of Sun Yat-sen University (Approval No. SYSU-IACUC-2023-001541) and conform to the Animal Research: Reporting of In Vivo Experiments (ARRIVE) guidelines.

For YAP/AKT-induced mouse ICC model, 30 μg of pT3-EF1a-YapS127A (Addgene) and 20 μg pT3-EF1a-HA-myr-AKT (Addgene) along with 2.85 μg Sleeping Beauty transposase plasmid (Addgene) were diluted in 2 ml of saline and injected into the lateral tail vein of liver-specific *Mettl5* cKO and WT mice within 5-7 seconds. All mice were sacrificed 4 weeks later and liver samples were collected for further analysis.

For TAM adoptive transfer model, clodronate liposome (1 mg/mouse, YEASEN) were injected into 3-4 weeks old *Mettl5* WT/cKO mice to deplete macrophages *in vivo*. YAP/AKT-induced mouse ICC model was established in C57BL/6 mice to provide TAMs. TAMs were isolated from tumors of the mice using anti-F4/80-MicroBeads (Miltenyi Biotec) for subsequent adoptive transfer. *Mettl5* WT or cKO mice were hydrodynamically injected with pT3-EF1a-YapS127A, pT3-EF1a-HA-myr-AKT and Sleeping Beauty transposase plasmids to induce ICC development. TAM transfer was performed by tail vein injection once a week for 5 doses (2 x 10^6^/mouse/dose). All mice were sacrificed after treatments and liver samples were collected for further analysis.

For *in vivo* T cell migration model, 1 × 10^7^ LTP-C9-G6 sg*NC* and sg*Mettl5* cells or LTP-C9-G6 sg*NC*+sh*NC* and sg*Mettl5*+sh*CXCL16* cells resuspended in 100 μL PBS were subcutaneously injected to the left and right flank of 6-8 weeks old NCG mice respectively. Tumor size was monitored and measured by Caliper every 3 days. Naive CD8^+^T cells isolated from spleen of C57BL/6 mice were activated by IL-2 (50 ng/ml, Peprotech) and CD3/CD28 Dynabeads (Thermo Fisher) at a bead to cell ratio of 1:1 for 3 days. When the tumor size reached 0.8 cm × 0.8 cm, the activated CD8^+^T cells (2 × 10^6^/mouse) were suspended in 100 μL PBS and adoptively transferred into NCG mice by tail vein injection. All mice were sacrificed 24 h after T cell injection. Tumor tissues from both flanks were collected for further analysis.

For orthotopic ICC model, 5 × 10^6^ LTP-C9-G6 wild-type or sg*NC* or sg*Mettl5* cells resuspended in 20 μL PBS were intrahepatically injected into the livers of C57BL/6, *Mettl5* WT/cKO or NCG mice respectively. NCG mice and *Mettl5* WT/cKO mice were sacrificed 4 weeks after tumor inoculation, and tumor samples were collected for further analysis. To evaluate anti-tumor effect of LNP-si*Mettl5* and PD-1 antibody in ICC, C57BL/6 mice were randomly separated into four treatment groups one week after tumor inoculation, including LNP-si*NC*+αIgG, LNP-si*Mettl5*+αIgG, LNP-si*NC*+αPD-1, LNP-si*Mettl5*+αPD-1. Lipid Nanoparticles (LNPs) loaded with si*NC/*si*Mettl5* were produced by Guangzhou Kelan Biotechnology. LNP-si*NC/Mettl5* (1 OD/mouse) and 100 μg αPD-1 (Bioxcell, RMP1-14) or αIgG (Bioxcell, 2A3) were administered to the mice by tail vein injection and peritoneal injection every 3 days respectively. All mice were sacrificed after treatments, and livers were collected for further analysis.

**scRNA-seq and bulk RNA-seq analysis**

**scRNA seqencing and analysis**

Fresh tumor samples were washed with PBS and cut into pieces. Human and mouse samples were then enzymatically digested using human tumor dissociation kit (Miltenyi, German) or mouse tumor dissociation kit (Miltenyi, German) respectively. Single cell suspensions were collected and loaded onto the Chromium single cell controller (10×Genomics) following the manufacture’s protocol. scRNA-seq, TCR VDJ and BCR VDJ libraries were constructed using 10×Genomics Chromium Single Cell 5’V(D)J Reagnet Kit (V2 chemistry, 10×Genomics) and sequenced on NovaSeq 6000 sequencer (Illumina).

Raw data were aligned and quantified by CellRanger (v6.0, 10×Genomics) against the reference genome GRCh38 and GRCm38 for human and mouse samples, respectively. Raw gene expression matrices merging, quality control, dimensionality reduction, and clustering were performed using the Seurat package (v 4.2.2). For mouse samples, genes covered by less than 3 cells, and cells that contained less than 500 unique molecular identifiers (UMIs) were filtered out. Subsequently, low-quality cells was filtered out based on three criteria: (1) the number of detected genes was more than 4000 genes or (2) fewer than 300 genes; (3) the percentage of mitochondrial genes was below 20. For human samples, genes covered by less than 3 cells were filtered out. Subsequently, low-quality cells was filtered out based on three criteria: (1) the number of detected genes was more than 6000 genes or (2) fewer than 200 genes; (3) the percentage of mitochondrial genes was below 20.

Cells with *METTL5/Mettl5* expression levels >1 in tumor cells are defined as *METTL5/Mettl5* positive cells. Cells expressing conflicting markers were manually removed to exclude potential doublet cells. The AUCell R pakage (v 1.20.2) was used to score the gene signatures.

**T cell receptor sequencing (TCR-seq) analysis**

The TCR sequencing data were aligned and quantified using the Cell Ranger VDJ pipeline with the genome GRCm38 as reference. scRepertoire (v1.8.0) was implemented to determine TCR clonal information and diversity based on the VDJC genes. We defined clone size of >100 and <=500 as “Hyperexpanded”, >20 and <=100 as “Large”, >5 and <=20 as “Medium”, >1 and <=5 as “Small”, and 1 as “Single”. Tumor-specific T cells were defined in two ways: (1) clones displaying an average expression level >0 for *Entpd1* and *Itgae* among CD8^+^ T cells, and (2) clones shared with “Exhausted T Cells” cluster ^3, 4^.

**B cell receptor sequencing (BCR-seq) analysis**

The BCR sequencing data were aligned and quantified using the Cell Ranger VDJ pipeline with the genome GRCm38 as reference. scRepertoire (v1.8.0) was implemented to determine BCR clonal information and diversity based on the VDJC genes.

**RNA sequencing and data analysis**

Total RNA was extracted using TRizol Reagent (Thermo Scientific). RNA libraries were constructed on the Illumina platform. Qualified reads were mapped to the human reference genome (GRCh37) by RSeQC with default parameters. Gene expression levels were standardized as TPM (Transcripts per million). Based on the median expression level of METTL5, patient samples were divided into High and Low group. Immune components were analyzed using CIBERSORT (v 0.1.0).

**Ribosome nascent-chain complex-bound mRNA sequencing (RNC-seq) and RNC-qPCR**

Analysis of translation efficiency of specific genes was performed as previously described ^5^. In brief, cells were incubated with 100 μg/ml cycloheximide at 37℃ for 15 min, followed by PBS washing and lysis with 1 mL lysis buffer (0.1% Triton X-100) diluted in ribosome buffer (RB buffer, 100 μg/ml cycloheximide, 2 mM dithiothreitol, 15 mM MgCl_2_, 20 mM HEPES-KOH and 200 mM KCl) on ice for 30 min. Cell lysates were centrifuged at 4℃, 16,200 g for 10 min. Then, 90% of the extraction was layered onto 11 mL 30% sucrose diluted in RB buffer, and ultra-centrifuged at 4℃, 185,000 g for 5 h to collect the RNC pellets that contain the polysome fractions. The 10% remaining extraction was used as input control. Next, total RNA were isolated from the input and RNC samples for sequencing and qRT-PCR.

**RNC-seq data analysis**

The cDNA library construction and sequencing were conducted by the Beijing Genomics Institute using BGISEQ-500 platform (BGI shenzhen, China) as previously described ^6^. The high-quality reads were aligned to the UCSC human reference genome (GRCh37/hg19) using Hisat2 tool. Gene expression levels were normalized using FPKM (Fragments Per Kilobase of transcript per Million mapped reads) method. Translational ratios (TR) were calculated using the following formula: TR = (FPKM in RNC-seq) / (FPKM in input RNA-seq). Genes with a fold change in translation efficiency of 2 or greater and a false discovery rate (FDR) of less than 0.05 were identified as differentially translated genes.

**Pathway and gene set enrichment analysis**

Gene Ontology (GO) Pathway enrichment analysis was performed with R package ‘clusterProfiler’ (v 4.7.1) to identify biological pathways enriched in specific gene lists. Pathways with adjusted *p*<0.05 were considered as significantly enriched. GSEA was performed with R package ‘clusterProfiler’ using the pathways from GSEA Human MSigDB Collections “C5” (https://www.gsea-msigdb.org/gsea/msigdb/).

**Ribosome profiling (Ribo-seq)**

Cells were incubated with 2 mg/mL harringtonine for 2 min, followed by 100 μg/mL cycloheximide at 37℃ for 5 min to terminate translation quickly, and washed twice by pre-chilled PBS containing cycloheximide (100 μg/mL). Cell suspension was collected by cell scraper and centrifuged at 4℃, 1000 rpm for 3 min. Cell pellet was lysed with lysis buffer (250 μl 1 M MOPS, 75 μl 1 M MgCl_2_, 150 μl 5 M NaCl , 50 μl 10 mg/ml cycloheximide , 2.5 ml 2 mg/ml Heparin , 50 μl 100 mM Benzamidine, 250 μl 10% Triton X-100, 100 μl 0.1 M PMSF, 25 μl 40U RNaseOUTTM Recombinant and 1550 μl DEPC) for 10 min on ice, followed by centrifugation at 4℃, 20,000 g for 10 min. 300 mL supernatant was incubated with 7.5 mL RNase I and 5 mL DNase I at room temperature for 45 min. Then, 10 mL RNase inhibitor was added to stop the digestion process. Size exclusion columns (illustra MicroSpin S-400 HRColumns, GE Healthcare) were equilibrated with 3 mL polysome buffer. The digested RNA was transferred to the equilibrated column, followed by centrifugation at 600 g for 2 min. The elution was subsequently mixed with 10 mL 10% SDS. RNA Clean & Concentrator-25 kit (Zymo Research) was used to extract the ribosome footprint fragments, followed by purification using magnet beads (Vazyme). Ribo-seq libraries were constructed using NEBNext Multiple Small RNA Library Prep Set for Illumina and subjected to sequencing ^6^.

**Methylated RNA immunoprecipitation sequencing (MeRIP-seq)**

Total RNA was extracted using TRizol Reagent (Thermo Scientific), treated with DNase I (Roche Diagnostics), and fragmented using RNA Fragmentation Buffer (100 mM Tris-HCl, 100 mM ZnCl_2_). After terminating the reaction with EDTA, a portion of the fragmented RNA was retained as input, while the rest underwent m^6^A immunoprecipitation (IP). Protein A magnetic beads (Thermo Scientific) and Protein G magnetic beads (Thermo Scientific) were washed twice with IP buffer (150 mM NaCl, 10 mM Tris-HCl [pH 7.5], 0.1% IGEPAL CA-630) and resuspended in 500 μL of IP buffer. 5 μg of anti-m^6^A antibody was added to the bead suspension, followed by rotation at 4°C for at least 6 hours. The antibody-bound beads were washed twice with IP buffer and resuspended in 500 μL of IP Reaction Buffer (composed of fragmented RNA, 5× IP buffer, and RNasin Plus RNase Inhibitor (Promega). The mixture was incubated at 4°C for 2 hours with gentle rotation. After incubation, the beads were washed twice with IP buffer, followed by two washes each with low-salt IP buffer (50 mM NaCl, 10 mM Tris-HCl [pH 7.5], 0.1% IGEPAL CA-630) and high-salt IP buffer (500 mM NaCl, 10 mM Tris-HCl [pH 7.5], 0.1% IGEPAL CA-630). RNA was eluted from the beads using RLT buffer from the RNeasy Mini Kit (QIAGEN) and purified according to the manufacturer's protocol. Libraries were prepared from input and IP RNA using the SMARTer Stranded Total RNA-Seq Kit - version 2 (Pico Input Mammalian, Takara/Clontech) and sequenced on the NovaSeq platform.

**ELISA**

2×10^5^ RBE and LTP-C9, sh*NC* and sh*METTL5/*sh*Mettl5* cells were seeded on 6-well plate. Supernatant was collected after 48 h for analysis. Mice tumor tissues were homogenized in RIPA buffer (Thermo Fisher) supplemented with protease inhibitor (Sigma-Aldrich) and phosphatase inhibitor (Sigma-Aldrich) on ice for 15 min, followed by centrifugation at 13,000 g for 15 min at 4℃. Supernatant was collected for analysis and normalized by protein concentration detected by BCA Protein Assay Kit (Thermo Fisher). CXCL16 concentration were detected by CXCL16 Mouse ELISA Kit (Boster Bio, EK0742) and CXCL16 Human ELISA Kit (Boster Bio, EK0741) according to the manufacturer’s protocols respectively.

Blood samples were collected from WT and cKO mice, and then centrifuged at 2000 rpm for 20 min. The serum was collected and analyzed for AST, ALT and CRP concentrations using ELISA kits (AST: MEIMIAN, MM44115M2; ALT: MEIMIAN, MM0260M2; CRP: Absin, abs552955) according to the manufacturer’s instructions.

**Polysome Profiling**

Upon reaching a cellular confluence of 95%, cells were incubated with 100 μg/mL cycloheximide at 37°C for 15 min to terminate translation, followed by PBS wash twice containing 100 μg/mL cycloheximide. Cells were collected and lysed in polysome cell extraction buffer (50 mM MOPS, 15 mM MgCl_2_, 150 mM NaCl, 100 μg/ml cycloheximide, 0.5% Triton X-100, 1 mg/ml heparin, 200 U/ml RNase inhibitor, 2 mM PMSF, and 1 mM benzamidine) on ice for 15 min. The cell lysates were centrifuged at 4°C, 13,000g for 10 min, and the supernatant was carefully layered onto a 10–50% gradient density sucrose solution followed by ultra-centrifugation at 4°C, 36,000 rpm for 150 min. After centrifugation, the supernatant was collected, fractionated at 0.75 mL/min using the BR-188 Density Gradient Fractionation system (Brandel) and monitored for the absorbance at 254 nm to quantify the polysome profiles. Monosome and polysome fractions were collected to isolate RNA, and detect *CXCL16/Cxcl16* mRNA levels.

**Single-base elongation and ligation-based PCR amplification (SELECT)**

SELECT was performed using Epi-SELECT m^6^A site identification kit according to manufacturer’s protocol (EPbiotek, China). Briefly, the designed probes flanking the m^6^A modification site at 18S rRNA position 1832 were annealed, followed by extension of a single base using SELECT DNA polymerase and nicking of the junction using SELECT Ligase. qPCR assays were then performed to measure the m^6^A level at the targeted site. The probes and primers used for SELECT in the study were as follow: Up Probe: tagccagtaccgtagtgcgtgCACCTACGGAAACCTTG; Down Probe: TACGACTTTTACTTCCTCTAcagaggctgagtcgctgcat; qRT-PCR Forward primer: 5’-ATGCAGCGACTCAGCCTCTG-3’; qRT-PCR Reverse primer: 5’-TAGCCAGTACCGTAGTGCGTG-3’.

**qPCR**

Total RNAs were isolated with Trizol reagent (Thermo Fisher) and then reverse transcribed to cDNA using the PrimeScript RT Master Mix Kit (Takara). The amplification of cDNA was performed using TB Green Premix Ex Taq II (Takara) according to the instructions from the manufacturer. The qPCR conditions were 95 ℃for 30 s; 95℃ for 5 s and 60℃ for 30 s; followed by 40 cycles of 95 ℃ for 15 s, 60 ℃ for 30 s and 95 for 15 s. All reactions were performed in triplicate. Relative mRNA expression was measured using GAPDH as an internal control. The primers used in this study were listed in **Supplementary Table 10**.

**Hematoxylin and Eosin (H&E) staining**

The H&E staining procedure was carried out as previously described ^5^. In brief, human or mouse tissues were fixed in 10% neutral-buffered formalin solution. After fixation, the tissues were embedded in paraffin and cut into 4 μm sections. Then, the slides were dewaxed in xylene, rehydrated through a series of decreasing ethanol concentrations. Subsequently, the sections were washed in water and stained with hematoxylin and eosin. Slides were scanned using a KF-PRO-020 Digital Slide Scanner (KFBio, China).

**Immunohistochemistry (IHC) staining**

Human and mouse tissue samples were collected, fixed in 4% paraformaldehyde and embedded in paraffin. After embedded, 4 μm sections were prepared and subjected to deparaffinization and rehydration. Antigen retrieval was performed using EDTA buffer (pH 6.0/8.0/9.0), and the 3% H_2_O_2_ solution was used to block the endogenous peroxidase activity for 10 min. The sections were further blocked using 20% goat serum, and incubated with specific antibodies diluted in 1% goat serum overnight at 4 ℃. The sections were then incubated with goat antirabbit HRP-conjugated secondary antibodies for 30 min at room temperature, followed by incubation with chromogen substrate solution provided by the Real Envision Detection System (DAKO). Finally, the sections were counterstained with hematoxylin to visualized cellular structure. The primary antibodies used in this study were listed in **Supplementary Table 11**.

**Western blot**

Western blot were performed as previously described ^5^. Briefly, cells were lysed in RIPA buffer (Thermo Fisher) supplemented with protease inhibitor (Sigma-Aldrich) and phosphatase inhibitor (Sigma-Aldrich). Protein concentration were assessed by BCA Protein Assay Kit (Thermo Scientific). 20-40 μg protein was separated by 10% SDS-polyacrylamide gel electrophoresis and electroblotted onto equilibrated on polyvinylidene difluoride membranes (Millipore). Membranes were blocked with 5% skim milk for 1 h at room temperature and then incubated with primary antibodies at 4°C overnight, followed by incubation with secondary antibodies for 1 h at room temperature. Finally, the protein signals were detected using the Amersham Imager 600 Imaging System (GE, USA). The antibodies used in this study were listed in **Supplementary Table 11**.

**T cell proliferation assay**

For autologous T cells proliferation assay, naive CD3^+^CD8^+^T cells were isolated from spleens of C57BL/6 mice and labelled with carboxyfluorescein succinimidyl ester (CFSE; 5 μmol/L; Invitrogen). Macrophages were isolated from tumors of WT and cKO mice and co-cultured with labeled CD3^+^CD8^+^T cells in the presence of CD3/CD28 dynabeads (Thermo Fisher) and recombinant IL-2 (50 ng/ml, Peprotech) for 3 days. CD3^+^CD8^+^T cells alone with or without dynabead stimulation was used as positive or negative control, respectively. Detection of T cell proliferation based on CFSE signals were performed by flow cytometry using Cytek Aurora (Cytek). The percentage of proliferating cells were calculated by FlowJo software (BD).

**T cell killing assay**

OT-1 CD8^+^T cells were isolated from spleen of OT-1 mice, and culture in the presence of CD3/CD28 dynabeads (Thermo Fisher) and recombinant IL-2 (50 ng/ml, Peprotech) for 3 days with or without TAMs isolated from WT/cKO mice (1×10^5^ OT-1 CD8^+^T cells, 1×10^5^ TAM per well). OVA-overexpressing LTP-C9 cells (0.5×10^5^ per well) were seeded into a 24-well plate. After 6 hours of adhesion, OT-1 CD8^+^T cells were added to and co-cultured with tumor cells at a ratio of 2:1. Following 72 hours of co-culture, apoptotic OVA-LTP-C9 cells was assessed using an Annexin V-FITC/PI apoptosis Detection kit (DOJINDO, AD10) according to the manufacturer’s instructions.

***In vitro* CD8^+^ T cell chemotaxis assay**

Human CD8^+^ T cells were isolated from peripheral blood mononuclear cells (PBMCs) of intrahepatic cholangiocarcinoma (ICC) patients using the Human CD8^+^ T Cell Isolation Kit (EasySep, STEMCELL Technologies). Mouse CD8^+^ T cells were isolated from the spleens of C57BL/6 mice using the Mouse CD8^+^ T Cell Isolation Kit (EasySep, STEMCELL Technologies). Conditioned medium (CM) was collected from RBE or LTP-C9-G6 cells cultured in DMEM supplemented with 10% FBS for 72 hours, followed by centrifugation at 500 g for 5 min to remove debris. For the chemotaxis assay, 1 × 10⁵ CD8^+^ T cells resuspended in 100 μl of FBS-free DMEM were seeded into the upper chamber of a 24-well Transwell insert with a 3-μm pore polycarbonate membrane (Corning), while 600 μl of CM was added to the lower chamber. Following incubation at 37°C for 24 hours, migrated cells were collected from the lower chamber and quantified using CountBright Plus Absolute Counting Beads (Thermo Fisher) according to the manufacturer's protocol and analyzed by flow cytometry .

**Flow cytometry**

Single cells were prepared and suspended in staining buffer (BD) with 1 μg/test Fc block (BD) for 10 min at 4 °C. Mouse samples were stained with anti-mouse fluorochrome-labeled antibodies : CD45 (30-F11, BD), CD11b (M1/70, BD), CD11c (N418, BD), F4/80 (BM8, BD), Gr1 (RB6-8C5, BD), Ly6C (HK1.4, Biolegend), Ly6G (1A8, BD), CD3 (145-2C11, BD), CD4 (GK1.5, BD), CD8α (53-6.7, BD), CD19 (1D3, BD), PD1 (EH12.2H7, BD), NK1.1 (PK136, BD), and CXCR6 (DANID2, Invitrogen). Cells were permeabilized in 100 μL of fixation/permeabilization solution (BD) for 20 min at 4°C, washed with 1×Perm/Wash buffer (BD), and stained intracellularly with IFN-γ (XMG1.2, eBioscience), CD206 (C068C2, BD) for 1 h at 4°C, followed by washing with 1×Perm/Wash buffer and fixation with 1% paraformaldehyde. Stained cells were analyzed on a Cytek Aurora flow cytometer (Cytek) and data were analyzed and illustrated using FlowJo software (BD). The antibodies used in this study were listed in **Supplementary Table 11**.

**Human specimens**

24 fresh ICC tumor specimens were collected for scRNA-seq analysis and 186 ICC tumors were used for bulk RNA-seq, and some of these samples were used for IHC staining and mIF staining. 17 ICC biopsy samples from patients who have received immunotherapy were used for IHC staining. All samples were collected with patient written consent at the First Affiliated Hospital of Sun Yat-sen University, Guangzhou, China, from 2012 to 2021. This study was conducted according to the principles of the Declaration of Helsinki and was approved by the Institutional Review Board of The First Affiliated Hospital of Sun Yat-sen University (Approval No. [2023]766).

**Multiplex immunofluorescence (mIF) staining and analysis**

4 μm human ICC tissue sections were used for mIF staining using Pano-7-color panel kit (Panovue) following manufacturer’s protocol. Slides were baked at 65℃ for 2 h, dewaxed in xylene, rehydrated through a decreasing ethanol series, fixed in 10% neutral formalin, followed by 3 wash with ddH_2_O for 1 min. Antigen retrieval was then performed at 100℃ for 15 min, and slides were washed with ddH_2_O for 1 min, 1×TBST for 2 min and then blocked with 10% goat serum (Bioss) for 10 min at RT. Primary antibodies used were: CD68 (1:1000, CST, 30 min, 37°C), C1QA (1:500, Novus, 30 min, 37°C), CD8 (1:200, abcam, overnight, 4°C), CXCR6 (1:200, Novus, 30 min, 37°C), IFN-γ (1:1000, abcam, 30 min, 37°C), and PD-1 (1:100, abcam, 30 min, 37°C). After primary antibody incubation, slides were washed with TBST 3 min for 3 times, followed by secondary antibody incubation (Panovue) for 10 min at RT. Slides were washed with TBST 3 min for 3 times and stained with Opal-650 (CD68, 1:1000, Panovue), Opal-700 (C1QA, 1:500, TG), Opal-540 (CXCR6, 1:200, Panovue), Opal-520 (CD8, 1:200, TG), Opal-570 (IFN-γ, 1:1000, TG)， Opal-620 (PD-1, 1:100, TG) for 10 min at RT. Nuclei were stained with DAPI 1:200 for 10 min (Panovue), washed and mounted with mounting medium (Panovue). Details on antibody information and fluorophore pairing for the panel described here are also listed in **Supplementary Table 11**.

All stained slides were scanned using TissueFAXS Spectra imaging system (TissueGnostics) at 10× and 40× magnification. Analysis was performed using StrataQuest software (v. 7.1.1.138; TissueGnostics). For analysis, tissues were first isolated from the image, and pseudocoloured multiplexed images were created. Cell nucleus of individual cell was idenified using a nuclear segmentation algorithm based on the DAPI image. The expression of CD68, C1QA, CD8, CXCR6, IFN-γ and PD-1 were computed for each cell. The number and proportion of positive cells for every marker was calculated by creating histogram and scatterplot of intensity.

**Statistical analysis**

Data are presented as mean ± SEM from at least three independent experiments. Comparisons between two groups were performed using the independent Student’s t test or Wilcoxon’s test, and one-way ANOVA was used to compare data in more than two groups by GraphPad Prism 8.3.0 (GraphPad Software). Kaplan-Meier survival analysis was conducted, with significance assessed using the log-rank test. *P* values are reported in the figures where appropriate. A two-tailed *p* value of <0.05 was regarded as statistically significant.

# Supplementary Figure 1


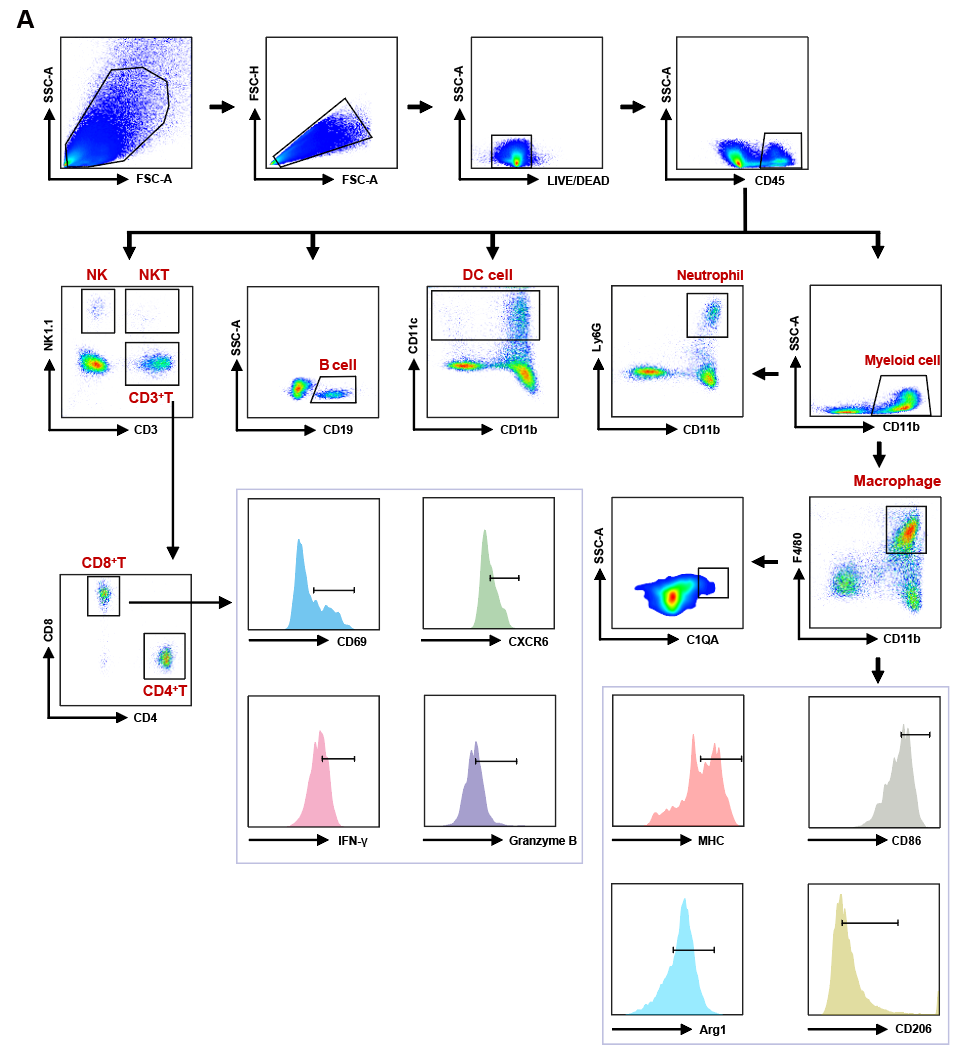


**Supplementary Figure 1. Gating strategy of flow cytometry analysis.**

# Supplementary Figure 2


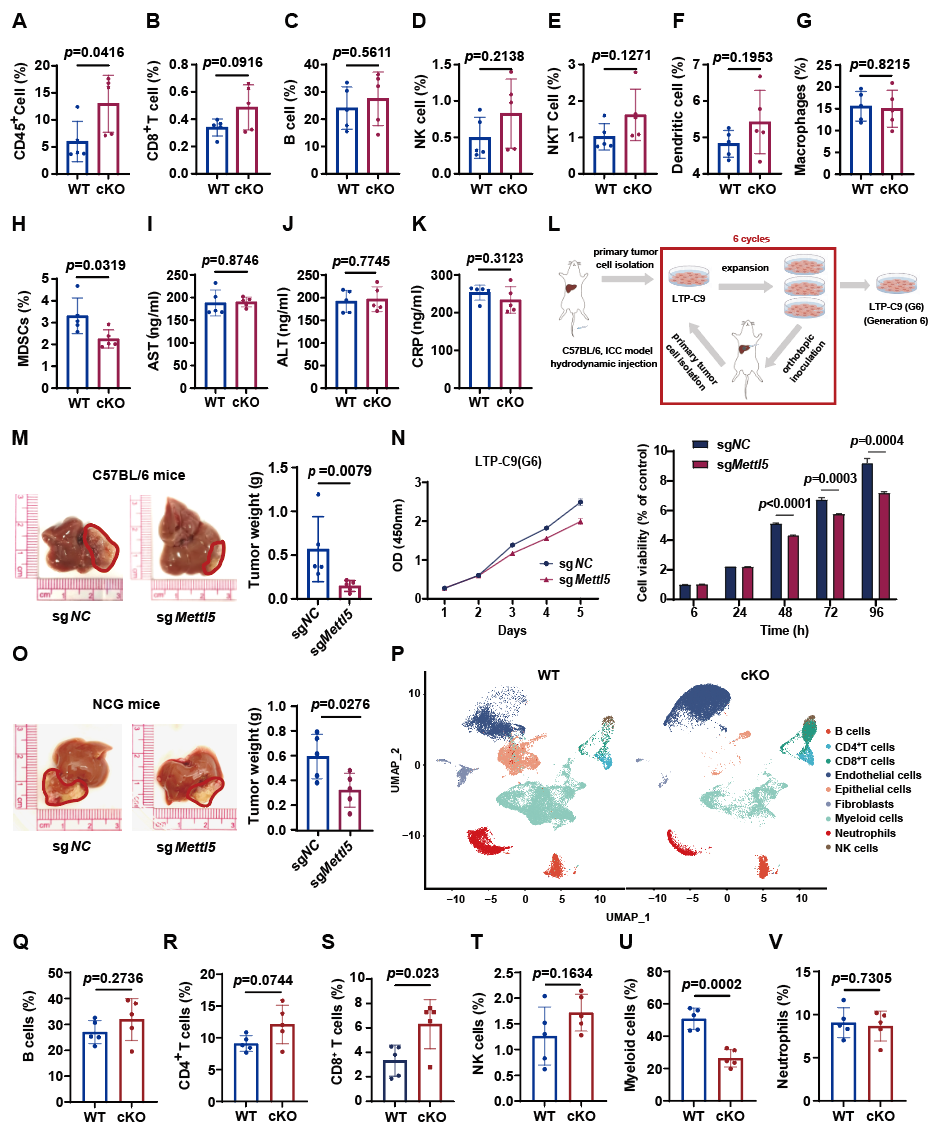


**Supplementary Figure 2. METTL5 shapes the liver immune microenvironment for ICC progression.** 6-8 weeks old *Mettl5* WT and cKO mice were euthanized for liver collection. (A-H) Percentage of liver-infiltrating CD45^+^ immune cells, CD8^+^ T cells, B cells, NK cells, NKT cells, dendritic cells (DCs), macrophages, and myeloid-derived suppressor cells (MDSCs) by flow cytometry analysis (n=5). (I-K) The aspartate transaminase (AST), alanine aminotransferase (ALT) and C-reactive protein (CRP) levels in the blood of WT and cKO mice. (L) The generation of mouse ICC cell line LTP-C9 Generation 6. (M) Representative gross images of mice livers and tumor weight. (N) Cell proliferation analysis of LTP-C9(G6) sg*NC* and sg*Mettl5* cells. (O) Representative gross images of mice livers and tumor weight. (P) scRNA-seq was performed on livers from 5 WT and 5 cKO mice. Based on lineage marker expression, cells were grouped into 9 clusters and illustrated as a UMAP plot. (Q-V) Percentage of B cells, CD4^+^ T cells, CD8^+^ T cells, NK cells, myeloid cells and neutrophils by flow cytometry analysis (n=5).

# Supplementary Figure 3


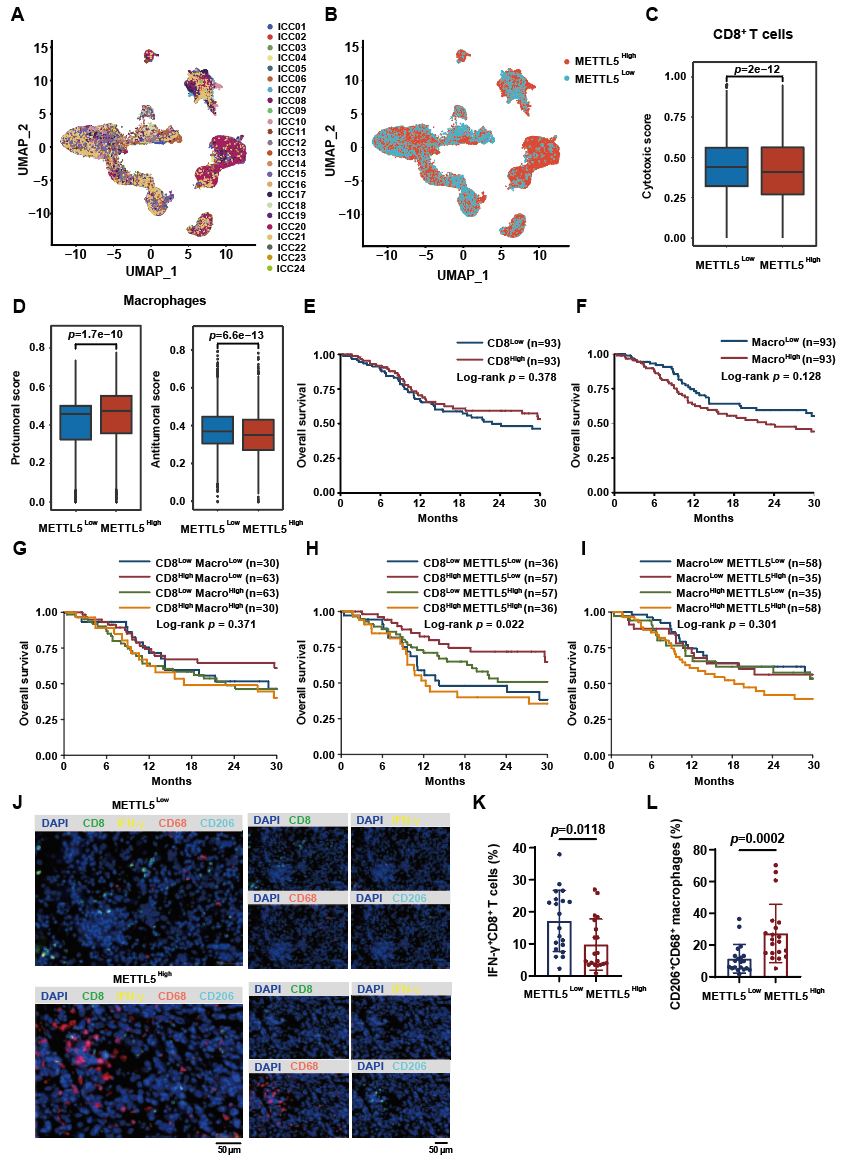


**Supplementary Figure 3. scRNA-seq analysis of human ICC samples.** (A-B) The UMAP plot of cells separated by individual patient (A) and METTL5 High and Low expression group (n=12 vs 12)(B). (C) Cytotoxic scores of CD8^+^ T cell. (D) The pro-tumoral and anti-tumoral score of macrophages in METTL5 High and Low expression group (n=12 vs 12). (E-I) Survival analysis of ICC patients. Patients were divided into different groups based on the median expression of METTL5, proportion of CD8 and macrophage from bulk RNA-seq data. (J) mIF staining of CD8, IFN-γ, CD68, CD206 and DAPI in METTL5 High and Low expression ICC paraffin tissues. (K-L) Statistical analysis of mIF staining of IFN-γ^+^CD8^+^ T cells and CD206^+^CD68^+^ macrophages in human ICC (n=20 vs 20).

# Supplementary Figure 4


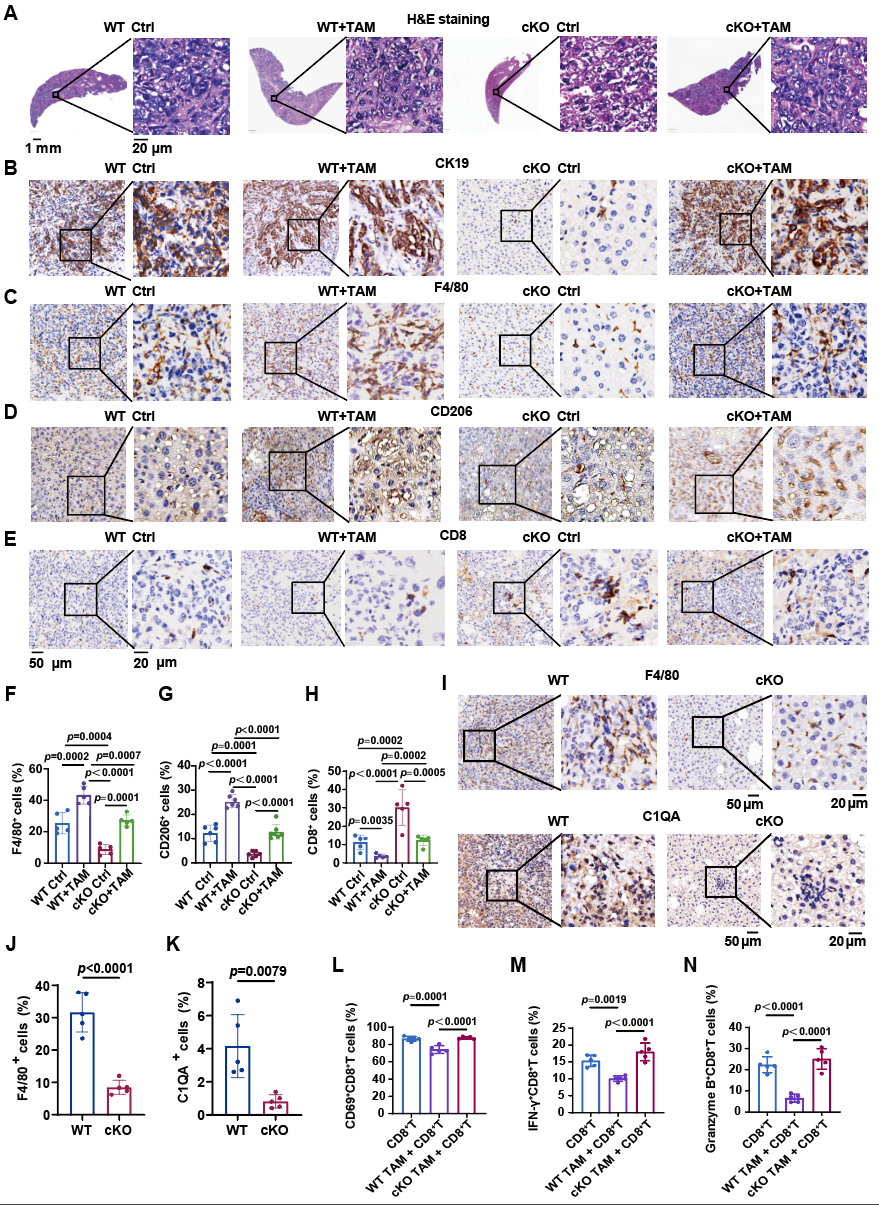


**Supplementary Figure 4. Immunosuppressive C1qa^+^TAM decreased in cKO mice.** (A-E) H&E staining (A) and IHC staining of CK19 (B), F4/80 (C), CD206 (D) and CD8 (E) of the mice livers. (F-H) Statistical analysis of IHC staining of F4/80^+^ (F), CD206^+^ (G) and CD8^+^ (H) cells (n=5). (I-K) IHC staining of WT and cKO mice livers showing F4/80 and C1QA expression and statistical analysis (n=5). (L-N) Proportion of CD69^+^CD8^+^T cells, IFN-γ^+^CD8^+^T cells and Granzyme B^+^CD8^+^T cells with or without co-culture with TAMs isolated from WT/cKO mice (n=5).

# Supplementary Figure 5


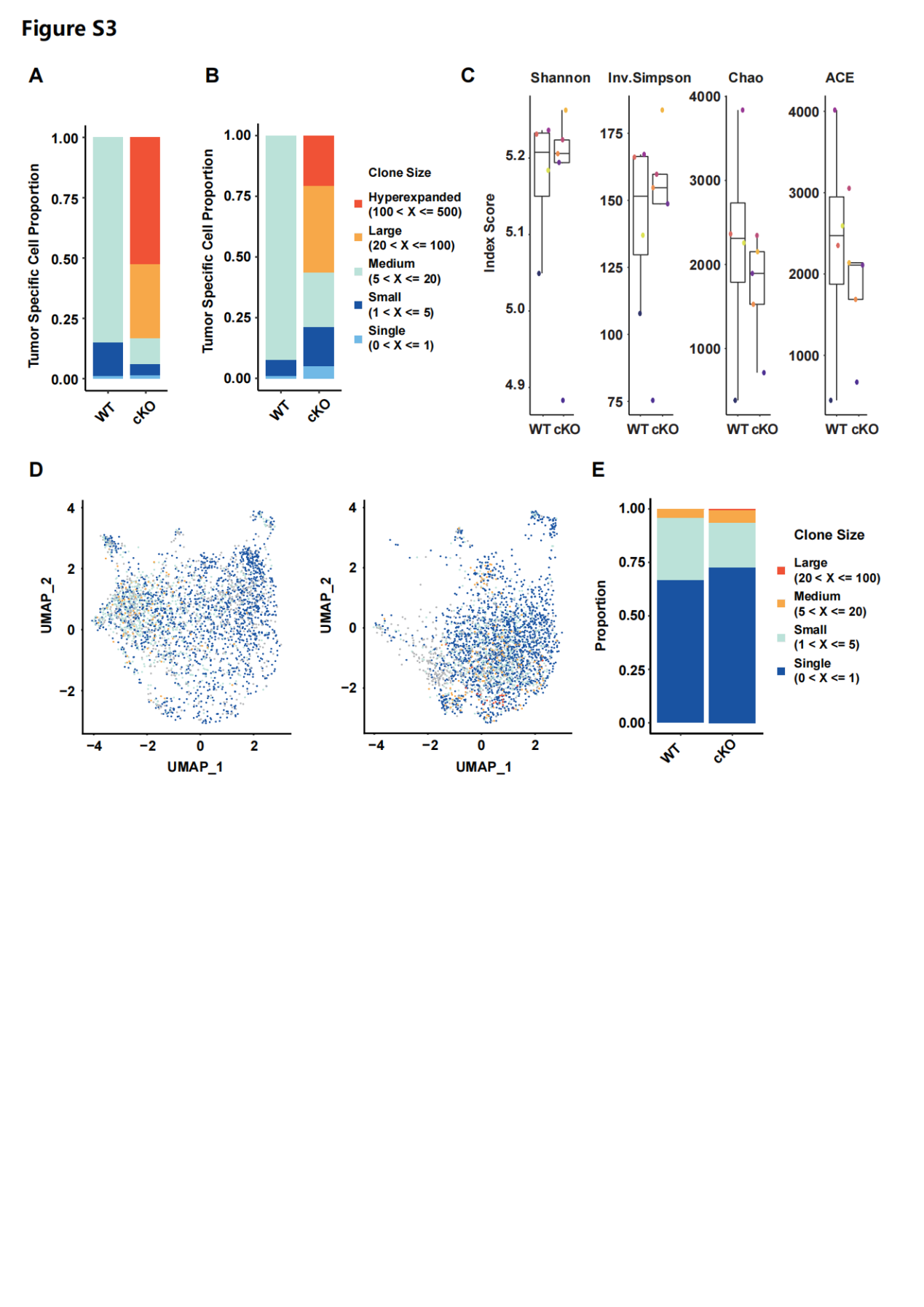


**Supplementary Figure 5. TCR clonal expansion instead of BCR is enhanced in cKO mice compared to WT mice.** (A-B) The proportion of expanded tumor-specific TCR clones in WT and cKO mice ^3, 4^. (C) The Shannon, Inv.Simpson, Chao and ACE index scores which represented BCR diversity in WT and cKO mice. (D) The UMAP plot showing the BCR clonal expansion of B cell sub-populations. (E) The proportion of expanded BCR clones in WT and cKO mice.

# Supplementary Figure 6


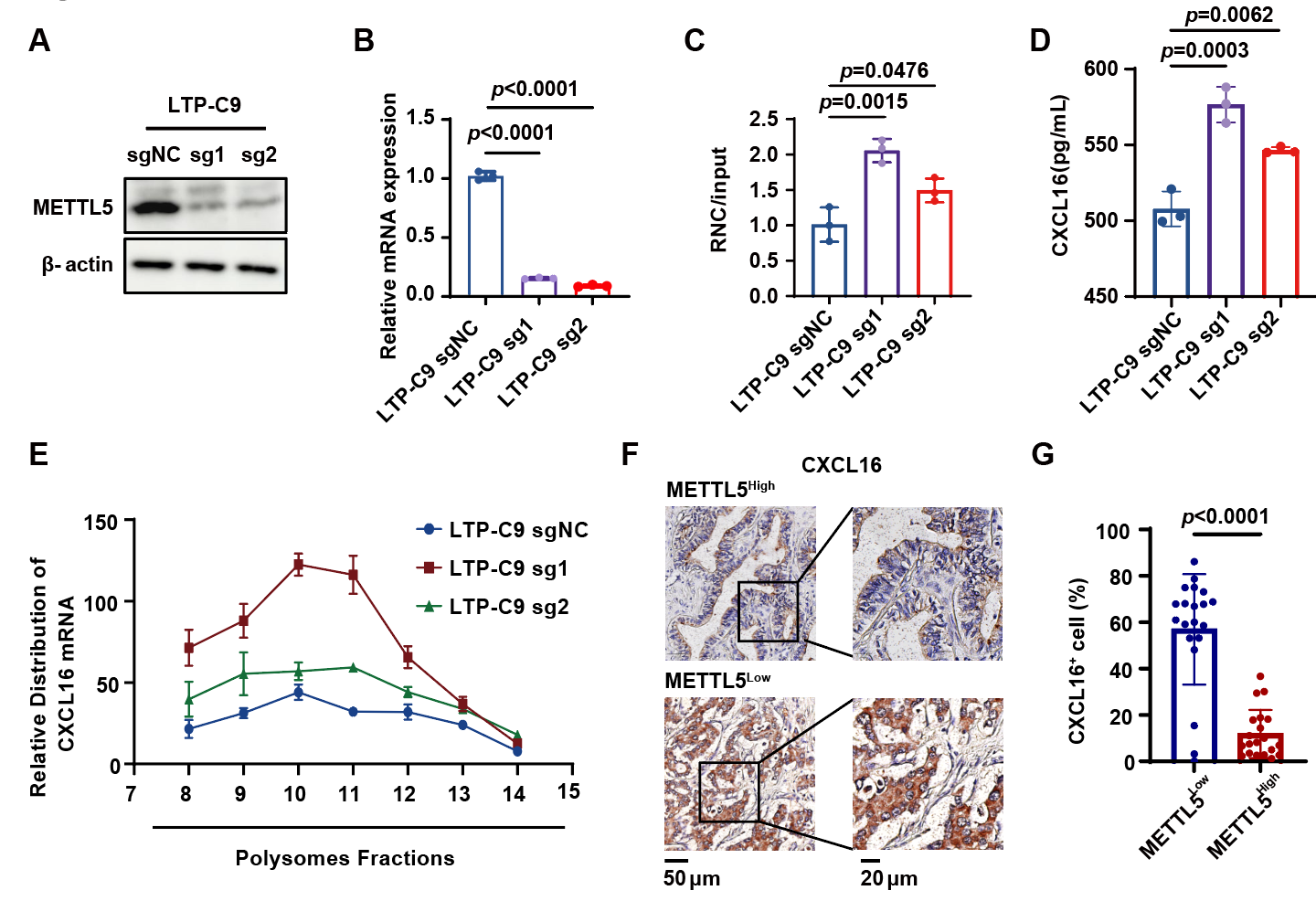


**Supplementary Figure 6. Knockout of *Mettl5* in LTP-C9 cells enhances the translation of *Cxcl16* mRNA.** (A-B) Western blot and QPCR analysis of METTL5 in LTP-C9 sg*NC* and sg*Mettl5* (sg1, sg2) cells. (C-E) RNC-qPCR (C), ELISA (D) and polysome profiling (E) showing the *Cxcl16* mRNA translation ratio, protein level and polysome fractions in LTP-C9 sg*NC* and two sg*Mettl5* cells (n=3). (F-G) IHC staining (F) and statistical analysis (G) of CXCL16 in METTL5 high and low expression human ICCs (n=20 vs 20).

# Supplementary Figure 7


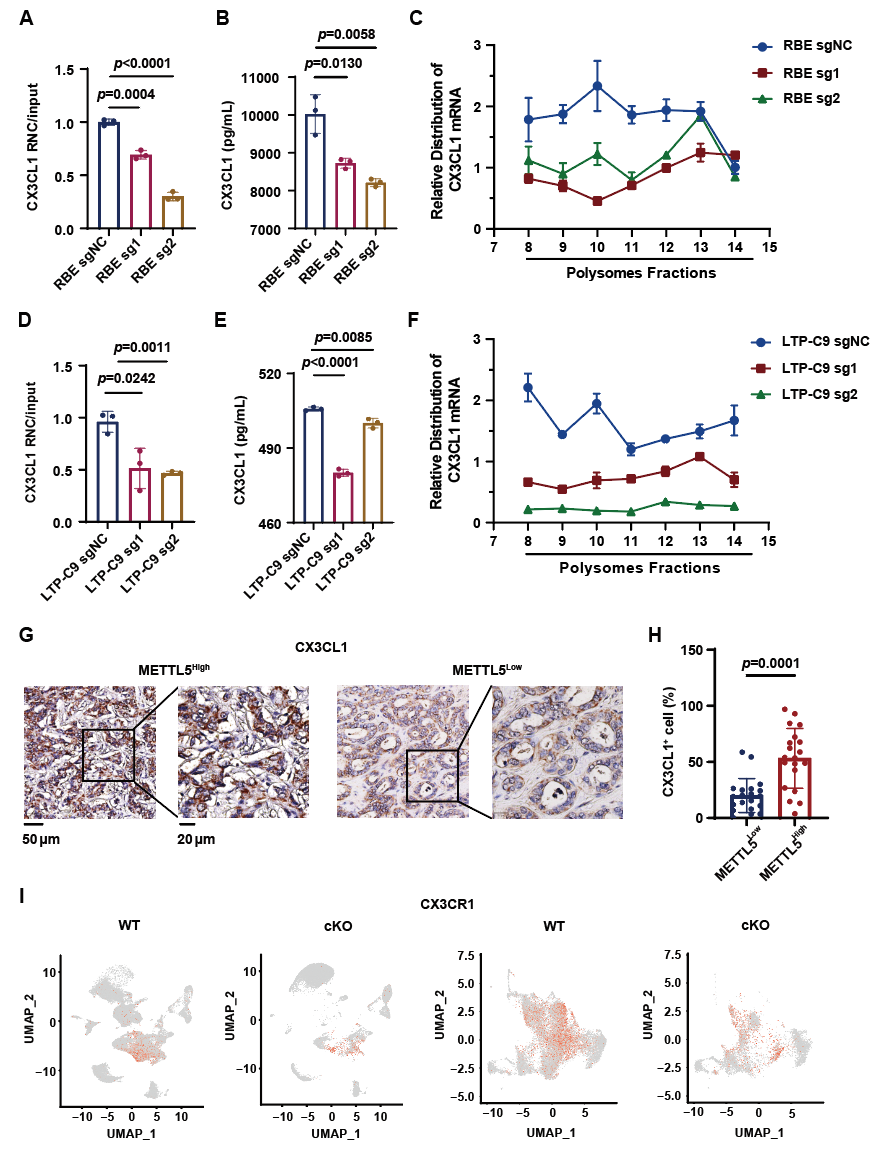


**Supplementary Figure 7. METTL5 regulated the mRNA translation of CX3CL1.** (A-C) RNC-qPCR (A), ELISA (B) and polysome profiling (C) showing the CX3CL1 mRNA translation ratio, protein level and polysome fractions in RBE sg*NC* and two sg*METTL5* cells (n=3). (D-F) RNC-qPCR (D), ELISA (E) and polysome profiling (F) showing the *Cx3cl1* mRNA translation ratio, protein level and polysome fractions in LTP-C9 sg*NC* and two sg*Mettl5* cells (n=3). (G-H) IHC staining (G) and statistical analysis (H) of CX3CL1 in METTL5 high and low expression human ICCs (n=20 vs 20). (I) The UMAP plot showing CX3CR1 positive cells in all clusters (left panel) and myeloid cell cluster (right panel).

# Supplementary Figure 8


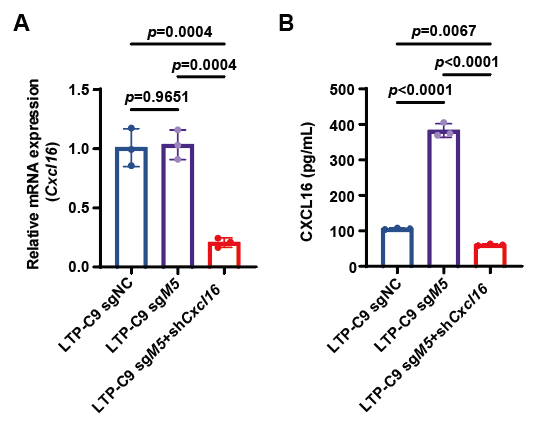


**Supplementary Figure 8. Knockdown efficiency of *Cxcl16*.** (A-B) The mRNA (A) and protein (B) level in LTP-C9 sgNC, sg*M5* and sg*M5*+sh*Cxcl16* cells.

# Supplementary Figure 9


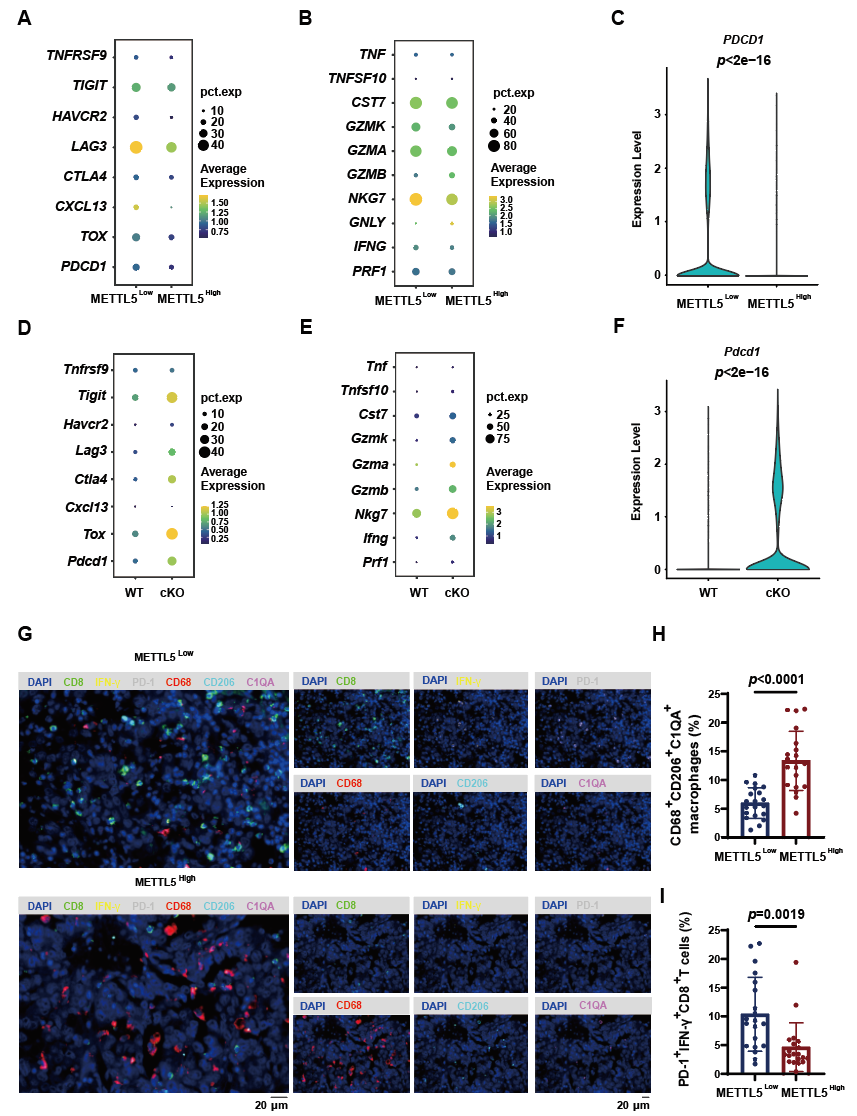


**Supplementary Figure 9. Expression of immune checkpoints, effector molecules and immune cell distribution in human and mouse ICC.** (A-C) Expression of immune checkpoints (A), effector molecules (B) and PD-1 (C) in human ICC samples (n=12 vs 12). (D-F) Expression of immune checkpoints (D), effector molecules (E) and PD-1 (F) in mouse samples (n=5 vs 5). (G-H) mIF staining and statistical analysis of CD8, IFN-γ, PD-1, CD68, C1QA, CD206 and DAPI in METTL5 High and Low expression human ICC paraffin tissues (n=20 vs 20).

# Supplementary Figure 10


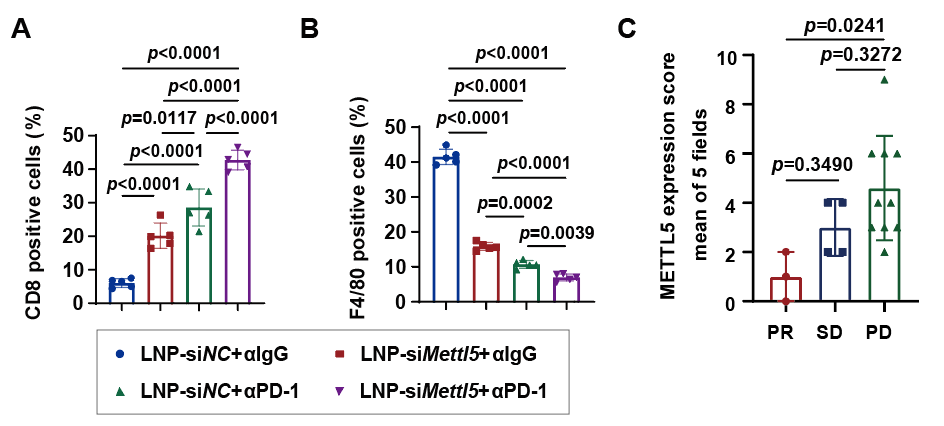


**Supplementary Figure 10. Statistical analysis of CD8 and F4/80 expression in IHC staining.** (A-B) Statistical analysis of CD8 (A) and F4/80 (B) expression. (C) Statistical analysis of METTL5 expression in 17 ICC biopsy samples before immunotherapy (n=3 PR, 4 SD and 10 PD patients).

Supplementary Table 1**. Baseline characteristics of ICC patients used for scRNA-seq**

| **Baseline characteristics** |  |
| --- | --- |
| Age(years) |  |
| <=60 | 17 (70.8%) |
| >60 | 7 (29.2%) |
| Sex |  |
| Male | 16 (66.7%) |
| Female | 8 (33.3%) |
| TNM |  |
| I-II | 9 (37.5%) |
| III-IV | 15 (62.5%) |

Supplementary Table 2**. METTL5 expression level of ICC patients included in scRNA-seq analysis**

| **Sample_id** | **METTL5** | **Sample_id** | **METTL5** |
| --- | --- | --- | --- |
| ICC01 | 0.509960253 | ICC13 | 1.369974033 |
| ICC02 | 0.314326296 | ICC14 | 0.753448471 |
| ICC03 | 1.049604051 | ICC15 | 1.224917703 |
| ICC04 | 1.086527498 | ICC16 | 1.002640429 |
| ICC05 | 0.658343461 | ICC17 | 1.31286274 |
| ICC06 | 0.707611751 | ICC18 | 0.994628135 |
| ICC07 | 0.821985249 | ICC19 | 0.508037475 |
| ICC08 | 1.480337497 | ICC20 | 1.10970974 |
| ICC09 | 0.649467295 | ICC21 | 0.57697613 |
| ICC10 | 1.112804362 | ICC22 | 0.796623996 |
| ICC11 | 0.444376825 | ICC23 | 1.192432371 |
| ICC12 | 2.059541064 | ICC24 | 0.869307341 |

* 24 samples were separated into METTL5 high and low expression group based on METTL5 median expression (0.9319677) (n=12 vs 12).

Supplementary Table 3**. Baseline characteristics of ICC patients used for bulk RNA-seq**

| **Baseline characteristics** |  |
| --- | --- |
| Age(years) |  |
| <=60 | 111 (59.7%) |
| >60 | 75 (40.3%) |
| Sex |  |
| Male | 106 (57.0%) |
| Female | 80 (43.0%) |
| TNM |  |
| I-II | 100 (53.8%) |
| III-IV | 86 (46.2%) |

Supplementary Table 4**. METTL5 expression levels of ICC patients included in bulk RNA-seq analysis**

| **Sample_id** | **METTL5** | **Sample_id** | **METTL5** | **Sample_id** | **METTL5** |
| --- | --- | --- | --- | --- | --- |
| ICC001T | 38.94825204 | ICC063T | 41.21747868 | ICC125T | 43.69503558 |
| ICC002T | 56.02045249 | ICC064T | 20.35895896 | ICC126T | 48.79621611 |
| ICC003T | 41.93744131 | ICC065T | 26.17024383 | ICC127T | 44.2471208 |
| ICC004T | 59.52005801 | ICC066T | 32.53765781 | ICC128T | 45.95475415 |
| ICC005T | 30.58237011 | ICC067T | 18.71665027 | ICC129T | 16.44388237 |
| ICC006T | 34.15010564 | ICC068T | 31.53665277 | ICC130T | 39.42908342 |
| ICC007T | 44.98695441 | ICC069T | 37.26582829 | ICC131T | 19.71298783 |
| ICC008T | 38.82650323 | ICC070T | 55.02901708 | ICC132T | 14.29858487 |
| ICC009T | 38.46489144 | ICC071T | 14.06153329 | ICC133T | 34.39811096 |
| ICC010T | 11.7612055 | ICC072T | 76.2914181 | ICC134T | 31.60016449 |
| ICC011T | 35.85296982 | ICC073T | 53.47726459 | ICC135T | 20.33910276 |
| ICC012T | 27.72010243 | ICC074T | 81.26027419 | ICC136T | 31.0848553 |
| ICC013T | 39.03011215 | ICC075T | 32.52608397 | ICC137T | 35.5144898 |
| ICC014T | 16.76695117 | ICC076T | 27.35453285 | ICC138T | 34.61016456 |
| ICC015T | 40.91802346 | ICC077T | 8.639200154 | ICC139T | 45.1402384 |
| ICC016T | 32.61602479 | ICC078T | 18.21690659 | ICC140T | 29.82744999 |
| ICC017T | 33.9382589 | ICC079T | 30.34864973 | ICC141T | 28.88003802 |
| ICC018T | 23.86515233 | ICC080T | 18.57113662 | ICC142T | 41.00986082 |
| ICC019T | 24.85726386 | ICC081T | 5.459669158 | ICC143T | 52.67730747 |
| ICC020T | 41.12066762 | ICC082T | 42.66376574 | ICC144T | 2.421641061 |
| ICC021T | 19.53084292 | ICC083T | 34.1815191 | ICC145T | 28.21994938 |
| ICC022T | 44.21288947 | ICC084T | 13.10885229 | ICC146T | 35.7656369 |
| ICC023T | 23.05054407 | ICC085T | 34.56823272 | ICC147T | 20.71431391 |
| ICC024T | 30.99078752 | ICC086T | 14.50074022 | ICC148T | 53.72440564 |
| ICC025T | 23.22401063 | ICC087T | 41.26091044 | ICC149T | 54.55159063 |
| ICC026T | 23.1613654 | ICC088T | 28.8055965 | ICC150T | 54.31984333 |
| ICC027T | 52.36827279 | ICC089T | 15.63172108 | ICC151T | 20.28353607 |
| ICC028T | 32.06805558 | ICC090T | 30.46293867 | ICC152T | 18.5817338 |
| ICC029T | 35.33226815 | ICC091T | 25.40336416 | ICC153T | 35.09165598 |
| ICC030T | 43.83152419 | ICC092T | 21.98176356 | ICC154T | 45.21720946 |
| ICC031T | 55.84636795 | ICC093T | 51.39183273 | ICC155T | 42.84555316 |
| ICC032T | 27.00443902 | ICC094T | 29.73060223 | ICC156T | 50.90590731 |
| ICC033T | 24.31985059 | ICC095T | 59.08124456 | ICC157T | 38.69518219 |
| ICC034T | 35.62446588 | ICC096T | 45.51052992 | ICC158T | 46.49301978 |
| ICC035T | 39.83982072 | ICC097T | 31.73887816 | ICC159T | 54.19265169 |
| ICC036T | 66.92160759 | ICC098T | 24.92379822 | ICC160T | 57.9160867 |
| ICC037T | 45.3366107 | ICC099T | 52.27111756 | ICC161T | 59.84292122 |
| ICC038T | 28.09142694 | ICC100T | 9.015663483 | ICC162T | 34.97173154 |
| ICC039T | 25.69876948 | ICC101T | 41.47662179 | ICC163T | 39.37613088 |
| ICC040T | 48.20710462 | ICC102T | 39.58091511 | ICC164T | 34.78480657 |
| ICC041T | 29.33593605 | ICC103T | 36.88366394 | ICC165T | 36.84735566 |
| ICC042T | 64.51167075 | ICC104T | 39.79429897 | ICC166T | 44.54698815 |
| ICC043T | 52.56199979 | ICC105T | 29.06473948 | ICC167T | 42.43555236 |
| ICC044T | 50.80128904 | ICC106T | 37.16871457 | ICC168T | 35.20666761 |
| ICC045T | 38.37558136 | ICC107T | 49.86603474 | ICC169T | 40.43631566 |
| ICC046T | 52.10377485 | ICC108T | 55.06212932 | ICC170T | 62.0166827 |
| ICC047T | 50.95219648 | ICC109T | 34.21665165 | ICC171T | 51.67238537 |
| ICC048T | 19.50295312 | ICC110T | 36.26821764 | ICC172T | 74.04225512 |
| ICC049T | 36.20886184 | ICC111T | 44.11136563 | ICC173T | 30.75956164 |
| ICC050T | 26.32464415 | ICC112T | 37.17568329 | ICC174T | 57.6465421 |
| ICC051T | 36.8897405 | ICC113T | 45.99542511 | ICC175T | 46.44431168 |
| ICC052T | 29.73774377 | ICC114T | 22.70917858 | ICC176T | 36.88523833 |
| ICC053T | 39.38562476 | ICC115T | 41.77179243 | ICC177T | 24.36174815 |
| ICC054T | 33.82543735 | ICC116T | 22.39676687 | ICC178T | 29.04293721 |
| ICC055T | 44.69828317 | ICC117T | 14.85582212 | ICC179T | 65.42974987 |
| ICC056T | 60.08008561 | ICC118T | 41.59800609 | ICC180T | 21.75614369 |
| ICC057T | 18.8590494 | ICC119T | 24.124327 | ICC181T | 34.63646086 |
| ICC058T | 45.08104393 | ICC120T | 32.82930654 | ICC182T | 24.71526071 |
| ICC059T | 25.46378984 | ICC121T | 36.06497388 | ICC183T | 36.07043271 |
| ICC060T | 58.72574015 | ICC122T | 28.00539122 | ICC184T | 56.55875837 |
| ICC061T | 13.79480857 | ICC123T | 52.17487482 | ICC185T | 28.91379888 |
| ICC062T | 59.38675358 | ICC124T | 30.11257763 | ICC186T | 8.535235971 |

* Based on the median expression level of METTL5 (35.8093), 186 patient samples were divided into High and Low group (n=93 vs 93).

Supplementary Table 5**. Multi-variate cox regression for CD8^+^T cells and RFS**

| **Baseline characteristics** | **HR** | **95% CI** | **P-values** |
| --- | --- | --- | --- |
| Age(years) |  |  |  |
| <=60 | 1.00 |  |  |
| >60 | 0.75 | (0.49, 1.15) | 0.186 |
| Sex |  |  |  |
| Male | 1.00 |  |  |
| Female | 0.96 | (0.64, 1.45) | 0.862 |
| TNM |  |  |  |
| I-II | 1.00 |  |  |
| III-IV | 3.19 | (2.11, 4.84) | 0.000 |
| CD8^+^T cells |  |  |  |
| Low | 1.00 |  |  |
| High | 0.65 | (0.43, 0.98) | 0.040 |

Supplementary Table 6**. Multi-variate cox regression for Macrophages and RFS**

| **Baseline characteristics** | **HR** | **95% CI** | **P-values** |
| --- | --- | --- | --- |
| Age(years) |  |  |  |
| <=60 | 1.00 |  |  |
| >60 | 0.77 | (0.51, 1.18) | 0.233 |
| Sex |  |  |  |
| Male | 1.00 |  |  |
| Female | 0.94 | (0.62, 1.42) | 0.765 |
| TNM |  |  |  |
| I-II | 1.00 |  |  |
| III-IV | 2.93 | (1.91, 4.48) | 0.000 |
| Macrophages |  |  |  |
| Low | 1.00 |  |  |
| High | 1.65 | (1.09, 2.51) | 0.019 |

Supplementary Table 7**. Multi-variate cox regression for CD8^+^T cells,_Macrophages and RFS**

| **Baseline characteristics** | **HR** | **95% CI** | **P-values** |
| --- | --- | --- | --- |
| Age(years) |  |  |  |
| <=60 | 1.00 |  |  |
| >60 | 0.76 | (0.50, 1.18) | 0.223 |
| Sex |  |  |  |
| Male | 1.00 |  |  |
| Female | 0.96 | (0.63, 1.46) | 0.851 |
| TNM |  |  |  |
| I-II | 1.00 |  |  |
| III-IV | 3.03 | (1.98, 4.65) | 0.000 |
| CD8-Macrophages |  |  |  |
| CD8^low^-Macro^low^ | 1.15 | (0.58, 2.30) | 0.690 |
| CD8^high^-Macro^low^ | 1.00 |  |  |
| CD8^low^-Macro^high^ | 1.98 | (1.19, 3.30) | 0.009 |
| CD8^high^-Macro^high^ | 1.30 | (0.68, 2.46) | 0.424 |

Supplementary Table 8**. Multi-variate cox regression for CD8^+^T cells_METTL5 and RFS**

| **Baseline characteristics** | **HR** | **95% CI** | **P-values** |
| --- | --- | --- | --- |
| Age(years) |  |  |  |
| <=60 | 1.00 |  |  |
| >60 | 0.78 | (0.51, 1.19) | 0.250 |
| Sex |  |  |  |
| Male | 1.00 |  |  |
| Female | 0.95 | (0.62, 1.45) | 0.801 |
| TNM |  |  |  |
| I-II | 1.00 |  |  |
| III-IV | 3.06 | (2.01, 4.65) | 0.000 |
| CD8-METTL5 |  |  |  |
| CD8^low^-METTL5^low^ | 1.99 | (1.07, 3.71) | 0.030 |
| CD8^high^-METTL5^low^ | 1.00 |  |  |
| CD8^low^-METTL5^high^ | 1.98 | (1.15, 3.41) | 0.013 |
| CD8^high^-METTL5^high^ | 1.65 | (1.04, 3.57) | 0.037 |

Supplementary Table 9**. Multi-variate cox regression for METTL5_Macrophages and RFS**

| **Baseline characteristics** | **HR** | **95% CI** | **P-values** |
| --- | --- | --- | --- |
| Age(years) |  |  |  |
| <=60 | 1.00 |  |  |
| >60 | 0.77 | (0.51, 1.18) | 0.237 |
| Sex |  |  |  |
| Male | 1.00 |  |  |
| Female | 1.00 | (0.66, 1.52) | 0.986 |
| TNM |  |  |  |
| I-II | 1.00 |  |  |
| III-IV | 3.01 | (1.96, 4.61) | 0.000 |
| METTL5-Macrophages |  |  |  |
| METTL5^low^-Macro^low^ | 1.00 |  |  |
| METTL5^high^-Macro^low^ | 1.11 | (0.58, 2.12) | 0.760 |
| METTL5^low^-Macro^high^ | 1.24 | (0.66, 2.34) | 0.496 |
| METTL5^high^-Macro^high^ | 2.11 | (1.23, 3.62) | 0.007 |

Supplementary Table 10**. Primer sequences used in this study**

| Species | Gene name | Forward Sequence（5’-3’） | Reverse Sequences （5’-3’） |
| --- | --- | --- | --- |
| Human | *CXCL16* | CCTATGTGCTGTGCAAGAGGAG | CTGGGCAACATAGAGTCCGTCT |
| Mouse | *Cxcl16* | GCAGGGTACTTTGGATCACATCO | AGTTCACGGACCCACTGGTCTT |
| Human/Mouse | *18S rRNA* | ACCCGTTGAACCCCATTCGTGA | GCCTCACTAAACCATCCAATCGG |
| Human | *METTL5* | GCCCAAGCTACTTCTGGAACAG | CCGATGCTAAGTACTCCACAACC |
| Mouse | *Mettl5* | GCGGTTGCAGATCTAGGATG | AATCCAACACACAACCCTGCT |
| Human/Mouse | *GAPDH/Gapdh* | AGGTCGGTGTGAACGGATTTG | GTAGACCATGTAGTTGAGGTCA |

Supplementary Table 11**. Detailed information of antibodies used in this study.**

| **Flow cytometry:** | | | | |
| --- | --- | --- | --- | --- |
| **Species** | **Antibody** | **Fluorophore** | **Clone** | **Vendor** |
| Mouse | Live/dead | Fixable Viability Stain 700 | - | BD Horizon |
| Mouse | CD45 | Alexa Fluor 532 | 30-F11 | eBioscience |
| Mouse | CD11b | APC-Cy7 | M1/70 | BD Pharmingen |
| Mouse | Gr-1 | BV750 | RB6-8C5 | BD Pharmingen |
| Mouse | Ly6G | PerCP-710 | 1A8 | eBioscience |
| Mouse | Ly6C | BV510 | HK1.4 | Biolegend |
| Mouse | F4/80 | FITC | BM8 | Biolegend |
| Mouse | CD11c | BV785 | N418 | Biolegend |
| Mouse | C1qa | primary antibody: ab155052, Rabbit anti-mouse; secondary antibody Anti-rabbit IgG (H+L), AF350 | A-11046(CAT) | Abcam; Invitrogen |
| Mouse | CD45 | PerCP-Cy5.5 | 30-F11 | BD Pharmingen |
| Mouse | CD3e | FITC | 145-2C11 | BD Pharmingen |
| Mouse | CD4 | BV650 | GK1.5 | BD Horizon |
| Mouse | CD8α | BV510 | 53-6.7 | BD Horizon |
| Mouse | IFN-γ | BV750 | XMG1.2 | eBioscience |
| Mouse | CXCR6 | APC | DANID2 | Invitrogen |
| Mouse | CD19 | APC-Cy7 | 1D3 | BD Pharmingen |
| Mouse | NK-1.1 | BV480 | PK136 | BD OptiBuild |
|  | | | | |
| **Western blot:** | | | | |
| **Species** | **Immunogen** | **Source** | **Dilution** | **Vendor** |
| Human/Mouse | METTL5 | Rabbit | 1:1000 | Proteintech |
| Human/Mouse | β-Actin | Rabbit | 1:1000 | CST |
|  | | | | |
| **IHC staining:** | | | | |
| **Species** | **Immunogen** | **Source** | **Dilution** | **Vendor** |
| Human/Mouse | METTL5 | Rabbit | 1:500 | Proteintech |
| Mouse | F4/80 | Rabbit | 1:500 | CST |
| Mouse | C1QA | Rabbit | 1:4000 | abcam |
| Mouse | CD8 | Rabbit | 1:500 | CST |
| Mouse | IFN-γ | Rabbit | 1:1000 | Abcam |
|  | | | | |
| **mIF staining:** | | | | |
| **Order** | **Antibodies** | **Dilutions** | **Opal fluorophores** | **Opal dilutions** |
| 1 | CD68 | 1:1000 | 650 | 1:300 |
| 2 | C1QA | 1:500 | 700 | 1:300 |
| 3 | CXCR6 | 1:200 | 540 | 1:300 |
| 4 | CD8 | 1:200 | 520 | 1:300 |
| 5 | IFNγ | 1:1000 | 570 | 1:300 |
| 6 | PD-1 | 1:100 | 620 | 1:300 |

**References**

1. Liu, H., X. Zeng, X. Ren, et al., Targeting tumour-intrinsic N(7)-methylguanosine tRNA modification inhibits MDSC recruitment and improves anti-PD-1 efficacy*. Gut,* 2022.

2. Peng, H., B. Chen, W. Wei, et al., N(6)-methyladenosine (m(6)A) in 18S rRNA promotes fatty acid metabolism and oncogenic transformation*. Nat Metab,* 2022. 4(8): p. 1041-1054.

3. Tan, J., W. Fan, T. Liu, et al., TREM2(+) macrophages suppress CD8(+) T-cell infiltration after transarterial chemoembolisation in hepatocellular carcinoma*. J Hepatol,* 2023.

4. Chen, S., C. Huang, G. Liao, et al., Distinct single-cell immune ecosystems distinguish true and de novo HBV-related hepatocellular carcinoma recurrences*. Gut,* 2023. 72(6): p. 1196-1210.

5. Zeng, X., G. Liao, S. Li, et al., Eliminating METTL1-mediated accumulation of PMN-MDSCs prevents hepatocellular carcinoma recurrence after radiofrequency ablation*. Hepatology,* 2023. 77(4): p. 1122-1138.

6. Dai, Z., H. Liu, J. Liao, et al., N(7)-Methylguanosine tRNA modification enhances oncogenic mRNA translation and promotes intrahepatic cholangiocarcinoma progression*. Mol Cell,* 2021. 81(16): p. 3339-3355 e8.
